# Androgyny and atypical sensory sensitivity associated with savant ability: a comparison between Klinefelter syndrome and sexual minorities assigned male at birth

**DOI:** 10.3389/frcha.2024.1356802

**Published:** 2024-11-08

**Authors:** Shintaro Tawata, Kikue Sakaguchi, Atsuko Saito

**Affiliations:** ^1^ Graduate School of Human Sciences, Sophia University, Chiyoda-ku, Tokyo, Japan; ^2^ Research Department, National Institution for Academic Degrees and Quality Enhancement of Higher Education (NIAD-QE), Kodaira-shi, Tokyo, Japan; ^3^ Faculty of Human Sciences, Sophia University, Chiyoda-ku, Tokyo, Japan

**Keywords:** autism spectrum disorder, Klinefelter syndrome, gender dysphoria, savant syndrome, synesthesia, atypical sensory sensitivity

## Abstract

**Introduction:**

The *extreme male brain* (EMB) theory, a major causal hypothesis of autism (ASD: autism spectrum disorder), attributes excess androgens during early development as one of the causes. While studies have generally followed the EMB theory in females at birth, the co-occurrence of ASD in males at birth has been observed in conditions that are assumed to be associated with reduced androgen action during early development, including Klinefelter syndrome (KS) and sexual minorities. ASD is also associated with atypical sensory sensitivity, synesthesia, and savant syndrome.

**Methods:**

In the present study, we examined adult KS individuals (*n* = 22), sexual minorities assigned male at birth (*n* = 66), and control males matched for age and educational background to those with KS [Exploratory analysis (control 1st): *n* = 36; Reanalysis (control 2nd): *n* = 583]. Participants completed a self-report questionnaire assessing sensory hypersensitivity/hyposensitivity, savant tendency (developed for the present study), synesthesia, and sexual aspects, including gender identity and sexual orientation.

**Results:**

The results of the exploratory analysis suggested that individuals with KS exhibited a higher tendency toward sensory hypersensitivity/hyposensitivity than the tendency exhibited by the controls. In the Reanalysis, sexual minorities were more likely to be synesthetes, and in both analyses sexual minorities exhibited a higher savant tendency and sensory hypersensitivity/hyposensitivity than the controls. Moreover, the gender dysphoric state was associated with phenotypes observed in individuals with ASD, such as synesthesia, savant tendency, and sensory hypersensitivity/hyposensitivity.

**Discussion:**

These results suggest a common physiological background among gender dysphoria, synesthesia, savant tendency, and atypical sensory sensitivity. Thus, androgynous features (reduced effects of sex steroids during early development) in males at birth may be partially related to the phenotype commonly observed in individuals with ASD. Based on the present results, we propose that the reduction of sex steroids during early development may lead to atypical neurodevelopment and be involved in the atypicality of external and internal sensory perception, and thus in the atypicality of self-concept integration, through the disruption of oxytocin and the gamma-aminobutyric acid system modulating the neural excitation/inhibition balance.

## 1 Introduction

Autism (ASD: Autism spectrum disorder/ASC: Autism spectrum condition) is characterized by impaired social interaction and communication and limited and repetitive forms of behaviors/interests, with a prevalence of approximately 1% in the general population (1). Baron-Cohen proposed “*The extreme male brain* (EMB) *theory of autism*” based on the fact that the prevalence of ASD is higher in males. The theory postulates that the discrepancy of strengths in cognitive domains in ASD can be explained by reduced *empathizing* (to identify another person's emotions and thoughts, and to respond to these with an appropriate emotion) and heightened *systemizing* (to analyze the variables in a system, to derive the underlying rules that govern the behavior of a system) (2). Excessive androgen activity during early development is thought to contribute to ASD (2, 3). The excellence of scientific abilities, including spatial cognition, intuitive physics, and preference for rule-based information, has also been attributed to high androgenic effects and the masculinization of the brain (2, 4, 5).

While the findings on ASD in females at birth are largely reported in accordance with the EMB theory, many findings on ASD in males at birth are inconsistent with this theory. In a study examining the association between ASD and physical characteristics, female participants with ASD had fewer feminine characteristics, a result consistent with the EMB theory, while male participants with ASD had fewer masculine characteristics, a trend opposite to the EMB theory (6). Moreover, atypical brain areas in females with ASD somewhat overlapped with areas that showed sex differences in neurotypical controls, in both grey and white matter volumes, suggesting that it is consistent with the EMB theory (7). On the other hand, this is not observed in males with ASD (7).

Furthermore, multiple chromosomal/genetic syndromes are associated with sex steroid deficiencies and the known risks of ASD (8). Klinefelter syndrome (KS) is a type of differences in sex development (DSDs) (9), with a high frequency of sex chromosome aneuploidy (major karyotype 47, XXY) occurring in approximately 1 out of 660 males at birth (10). The typical phenotypes of KS include infertility, hypergonadotropic hypogonadism, dwarf testes, and gynecomastia (10). Low testosterone levels are prominent after puberty, although it has been suggested that some patients may have reduced androgen activity since early development (11–13). Individuals with KS often have neurodevelopmental disorders, such as ASD and attention-deﬁcit/hyperactivity disorder (ADHD) (14, 15). ASD is estimated to occur in approximately 30%–50% of the KS population (10). Further, studies using the autism spectrum quotient (AQ)—which measures the characteristics of ASD traits within the typical developmental range—have found that individuals with KS have higher AQ scores than those of control males (16, 17). Individuals with KS generally identify themselves as men, as do typical men, and have a gynephilic sexual orientation. However, there exist cases of gender dysphoria (GD) (18, 19). Furthermore, a systematic review reporting the prevalence of KS among male-to-female (MTF) transgender individuals assigned male at birth found that 9/1,013 (0.88%) had KS, suggesting a higher prevalence of KS among MTF transgender individuals (20). Fisher et al. (17), who studied KS patients attending the sexual medicine and men's department of a university hospital, reported that the patients had a lower GD tendency than that exhibited by MTF transgenders, but a higher tendency than that exhibited by control men, and KS’ propensity to GD may be mediated by ASD traits.

ASD also tends to co-occur in individuals belonging to sexual minorities, who are characterized by atypical sexual orientations and gender identities (8). A meta-analysis of 25 studies on transgender and other gender-nonconforming individuals (*n* = 8,662) estimated an ASD prevalence of approximately 11% (21). In the general theory of sexual differentiation of the brain, it is a common assumption that exposure to sex steroids such as androgens, genes, and chemicals, which are atypical of an individual's genetic sex in early development, leads to a mosaic pattern of sex differentiation (22). This results in a range of anatomically and psychologically neutral characteristics, which may include tendencies towards homosexuality and transgender identity. For sexual minorities assigned males at birth, the ratio of the length of the index finger to the length of the ring finger (2D:4D) and masculinity at play in childhood, indirect measures of early developmental androgen exposure, were more feminized in gay men and MTF transgenders than in control men [2D:4D in gay, (23); 2D:4D in MTF (24), masculinity at play in gay, (25)]. Although a meta-analysis reported no difference from controls concerning 2D:4D in gay men (26), indirect evidence, including increased sex nonconformity and birth order, suggests that androgen action may be reduced during early development in gay men who are receptive partners during intercourse (a bottom anal sex role) (27).

As mentioned above, many findings in males at birth do not support the early androgen excess (EMB) theory in ASD, and indicate that ASD is more likely to co-occur with atypical sexual differentiation, suggesting that androgynous characteristics (reduced early androgen action in males at birth) rather than extreme male brains explain the emergence of ASD. The present study focused on KS, in which a reduction in androgen action at early developmental stages is observed in a part of the population, and on sexual minorities assigned male at birth, in whom a reduction in androgen action at early developmental stages is assumed due to indirect circumstantial evidence. A comparison between the two groups was expected to provide clues for examining the mechanisms by which reduced androgen action affects the appearance of ASD-related symptoms.

In addition, atypical sexual differentiation and ASD traits may be associated with cognitive strength such as *savant syndrome* (the situation when an individual has outstanding talents despite their handicaps, such as neuropsychiatric disorders) and giftedness, through sensory characteristics such as *synesthesia* (a perceptual phenomenon in which the stimulation of one sense unconsciously triggers another) and atypical sensory sensitivity (8). Both synesthetes and individuals with ASD have increased sensory hypersensitivity/hyposensitivity compared to that in controls (28). Moreover, among various etiologies postulated for the development of savant syndrome (29), *the enhanced perceptual functioning* (EPF) model postulates that enhanced sensory processing develops savant ability (30). Baron-Cohen et al. (31) also posited that atypical senses promote attention to detail, which develops into systemizing and leads to savant ability. Hughes et al. (32) suggested that ASD individuals who are savants have higher scores on sensory hypersensitivity/hyposensitivity compared to the scores of ASD individuals who are not savants. Therefore, we examined the relationship between cognitive strength associated with ASD traits (savant tendency) and atypical sensory sensitivity, which was assumed to be the cause of ASD traits. To our knowledge, this study is the first to examine KS as a comparative subject from this perspective.

We conducted an exploratory study of sensory hypersensitivity/hyposensitivity, savant tendency, synesthesia, and sexual aspects among individuals with KS, sexual minorities assigned male at birth, and control males (control 1st and control 2nd group) with an online self-report questionnaire. For the sexual minority group, we recruited individuals who self-identified as “sexual minorities” as eligible to participate (the term “sexual minority” generally refers not only to sexual orientation minorities but also to gender minorities in Japan). The control males do not have a KS diagnosis and not belonging to a sexual or gender minority group. The control 2nd group was conducted after the analysis of the other groups, so some of the results obtained among KS, sexual minority, and the control 1st were reanalyzed.

## 2 Materials and methods

The study was registered on OSF (https://osf.io/kyg76) before the reanalysis (before the control 2nd group was recruited).

### 2.1 Recruitment of participants for KS and sexual minorities

For participants with KS and sexual minorities, we asked the relevant hospitals, clinics (urology and psychiatry), and support groups in Japan to distribute and share announcements to recruit participants for the study. We also recruited through word of mouth and SNS. Interested individuals accessed research websites brought by these announcements. They became eligible for the study by accessing the survey page and agreeing to participate.

### 2.2 Procedure

For individuals with KS and sexual minorities, the survey was conducted from July 13, 2021, to February 28, 2022, using SurveyMonkey (https://jp.surveymonkey.com) with a web-based questionnaire. Although the website used to access the survey pages was the same for KS and sexual minority groups, the surveys were conducted separately for either group. The survey page of sexual minorities, due to the assumption that people might not be familiar with KS, was titled as “Survey on Cognitive Characteristics in Sexual Minorities”. At the end of the questions, the title and aim of the study were debriefed and the consent on the use of data was reconfirmed.

For the control 1st group, a survey was conducted from November 15 to 27, 2021 using Surveroid (https://surveroid.jp), a self-administered survey tool provided by Marketing Applications, Inc. After screening, we selected men with no history of KS or hormone administration who were matched to the KS group in terms of age and educational background. Due to the assumed unfamiliarity with KS, the survey was titled as “Questionnaire on Individual Differences in Cognitive Characteristics in Adults”, and consent was obtained again after debriefing at the end of the question.

For the control 2nd group, the survey was conducted from February 8 to 10, 2023 among male monitors using a survey service (https://insight.rakuten.co.jp/) provided by Rakuten Insight Inc. After screenings were conducted (max *n* = 10,000), only males with no history of chromosomal or endocrine disorders or hormone administration and whose age and educational background corresponded to those of individuals with KS participated in the survey. Due to the assumed unfamiliarity with KS, the questionnaire was administered as “Questionnaire on Individual Differences in Cognitive Characteristics in Adults”, and consent was obtained again in the post-question debriefing.

### 2.3 Ethical considerations

This study was approved by the Ethics Committee on Research Involving Human Subjects of Sophia University (approval numbers: 2020-101, 2021-155, 2022-051, 2022-100, 2022-127) and the Ethics Committee of the National Institution for Academic Degrees and Quality Enhancement of Higher Education (NIAD-QE).

### 2.4 Participants

**Figure 1:**
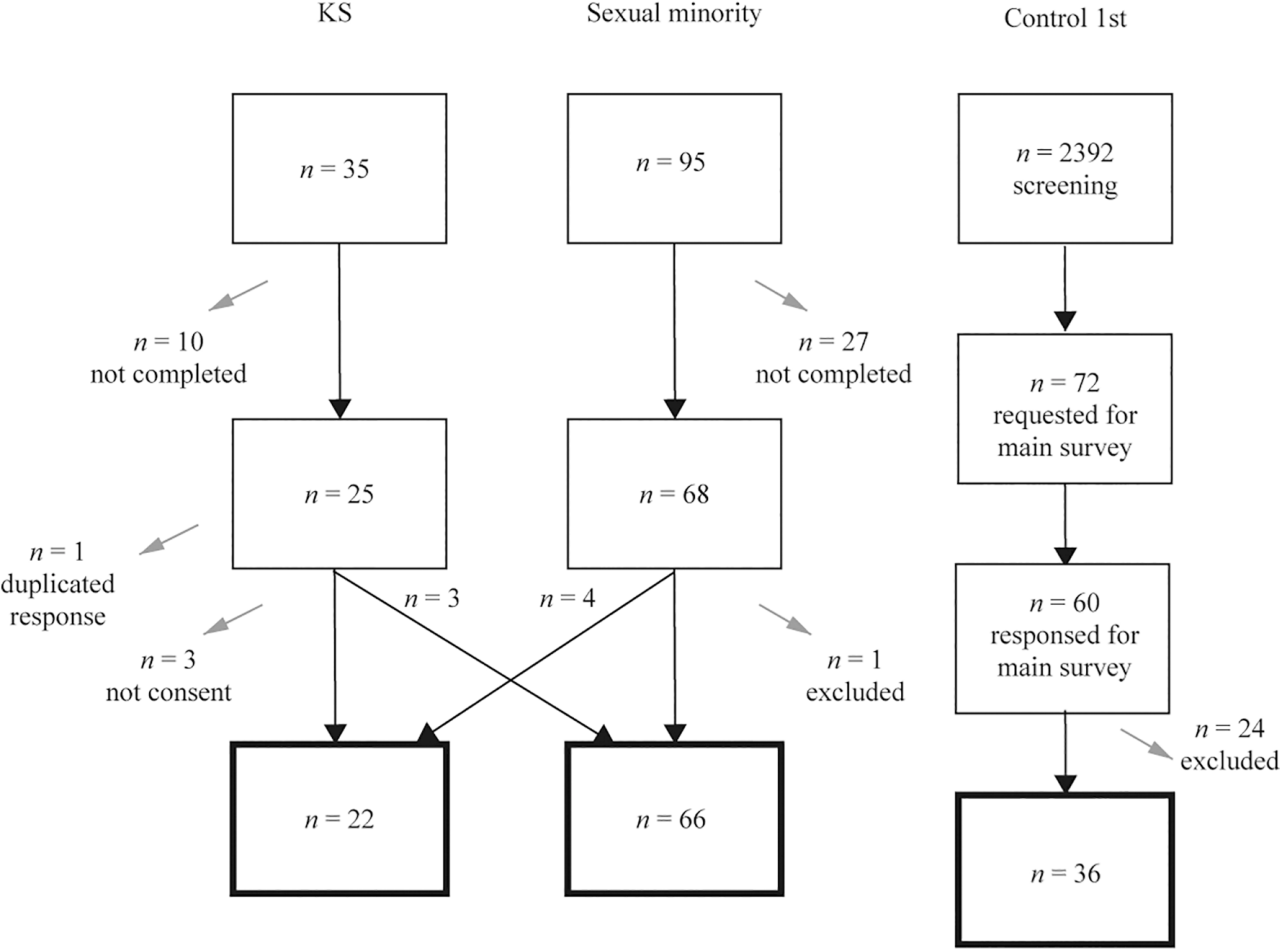
Flow of the analytic subjects in Exploratory analysis. Gray arrows indicate dropout from the survey. The squares with a thick black line at the bottom indicate the final subjects of the analysis.

The sample consisted of adults whose sex assigned at birth was male. The flow chart of the participants with sample sizes in Exploratory analysis is shown in Figure 1.

Thirty-five participants responded to the form for KS, and 25 completed the form. Of these, those who did not provide consent (*n* = 3), reported no diagnosis of KS (*n* = 3), and were considered to have double responses (*n* = 1; the older response was deleted) were excluded. Those who responded to the form for sexual minorities (*n* = 4) were added, leaving a total of 22 in the KS group.

Ninety-five people responded to the form for sexual minorities, and 68 completed the form. Of these, those who answered that they were diagnosed with KS (*n* = 4) and those who were presumed not to be male at birth based on their free answers (*n* = 1) were excluded. Those who answered the form for KS (no diagnosis of KS) (*n* = 3) were included in the sexual minority group, leaving a total of 66 participants in the sexual minority group.

The control 1st were screened (*n* = 2,392), and men with no history of KS or hormone administration, matched in age and educational background with the KS group (*n* = 72), were selected to participate in the study. Sixty men participated in the study, and we excluded those who did not agree at the beginning or withdrew from the study (*n* = 10) and those who did not agree at debriefing (*n* = 14); 36 men were included in the control 1st group.

In the control 2nd group, a screening was conducted on up to 10,000 individuals, and the study was concluded as soon as the number of participants who met the criteria reached 600. The researchers did not have information on the actual number of individuals who underwent the screening. 600 participants completed the survey. Of these, 17 were excluded because they identified themselves as sexual minorities [lesbian, gay, bisexual, transgender (LGBTs)], leaving 583 respondents in the control 2nd group.

### 2.5 Measures

A questionnaire with the following content was administered to the KS, sexual minority, control 1st groups (see OSF for details). The control 2nd was asked only the questions as follows: (2) synesthesia, (3) savant tendency, and (6) sensory hypersensitivity/hyposensitivity. At screening in the control 2nd, participants were asked whether they were male at birth, whether they had any diagnosis (for example, chromosomal aneuploidy or endocrine disorder), whether they had been administered hormones, their age, and their educational background. Participants in the control 2nd were also asked whether they considered themselves a sexual minority (LGBTs).

(1) **Preliminary questions**

The participants were asked whether they had been diagnosed with KS and had a history of hormone treatment and administration. Participants with KS were also asked about their chromosome type and how KS was discovered.

(2) **Synesthesia**

The participants were given a simple definition of synesthesia, as follows: “the spontaneous perception of multiple senses from a single object, such as color in letters, color in smells, taste in sounds, and sound in pictures”. The participants were then asked whether they have experienced synesthesia and were asked to describe types of synesthesia, if any, and whether and how it had changed with age or hormone administration.

(3) **Savant tendency**

Items were developed based on the domains of savant syndrome listed by Hughes et al. (32) and specific cases known to the authors. The items were accompanied by the instruction, “Answer what you are good at compared to others, and what special skills or abilities you have that others may not”. The 19 items consisted of a 5-point scale: “not true”, “somewhat not true”, “undecided”, “somewhat true”, and “true”. The items included “I am good at learning new languages”, among others. Responses were scored on a scale of 1–5 and the total score was calculated. Additional, more detailed questions were asked on several items (Supplementary S1).

(4) **Sexual aspects**

**Gender dysphoric state:** The participants were asked to answer “Yes”, “No”, or “Other (free description)” to the question “Do you feel uncomfortable being a male at this moment?” The participants who had been administered hormones were also asked whether they experienced any changes before and after the administration and were asked to describe the changes in detail.

**Diagnosis of GD/GID:** A history of GD or gender identity disorder (GID) diagnosis was asked. GD is the diagnostic name used in DSM-5, and GID was used before that. In GD, not only those who consistently desire to be of the opposite gender, but also those who desire to be of a different gender from the gender assigned at birth, such as nonbinary, are included in the diagnosis.

**Kinsey score (sexual orientation):** The participants were asked to indicate their current sexual orientation using the Kinsey scale (33), with the target gender of sexual interest indicated as “female: 1” and “male: 7” as the two extremes, and “female and male of the same level: 4” as the center, and “none of the above/asexual: X”. Sexual orientations were asked on three dimensions comprised of “romantic partner”, “fantasy”, and “actual sexual behavior”. The average scores of the three dimensions were calculated and used as the Kinsey scores. Answers such as “none of the above/asexual: X” were excluded from the average calculation. Participants who had been administered hormones were asked whether they had experienced any changes in their sexual orientation through hormone use.

**Masculinity of the play:** The participants were asked to select all items that applied to their favorite childhood games and toys. Sato and Koizumi (34) developed a battery of items for Japanese preschoolers. The questionnaire consisted of 13 items preferred by boys and 12 items preferred by girls; the masculinity of these items was considered to reflect fetal androgen exposure**.** In the present study, we excluded gender-neutral items based on a study by Sakaguchi et al. (25) and used eight items preferred by boys and girls each (Supplementary S1). The masculinity of play was calculated as follows: (number of boys’ play items chosen−number of girls’ play items chosen)/(number of boys’ play items chosen + number of girls’ play items chosen). Values ranged from −1 to 1, with a larger value corresponding to a more masculine preference.

**Cross-dressing:** The participants were asked whether they had ever preferred to wear women's clothing using a 5-point scale: “not true”, “somewhat not true”, “undecided”, “somewhat true”, and “true”. Responses were scored on a scale of 1–5. The participants were asked whether they experienced any changes with age or hormone administration.

**Male friends in childhood:** The participants were asked whether they had more male friends than female friends in elementary school and pre-school, using a 5-point scale: “not true”, “somewhat not true”, “undecided”, “somewhat true”, and “true”. The responses were scored on a scale of 1–5.

(5) **Demographic data**

Age, handedness, educational background, history of mental illness, diagnosis of developmental disorder, other medical history, height and weight, and marital status with a woman were asked. Each participant's body mass index was calculated based on height and weight.

(6) **Sensory hypersensitivity/hyposensitivity**

To measure atypical sensory sensitivity, we used the Japanese version of the Glasgow Sensory Scale (GSQ) (35) created by Robertson and Simmons (36). The GSQ consists of 42 items in seven modalities (visual, auditory, gustatory, olfactory, tactile, vestibular, and proprioceptive): three items for hypersensitivity and three items for hyposensitivity. The five responses were “Never”, “Rarely”, “Sometimes”, “Often”, and “Always”. The items included “Do you ever find certain noises or pitches unpleasant?” The responses were scored from 0 to 4, and the total score ranged from 0 to 168.

### 2.6 Statistical analyses

The alpha coefficient was calculated to examine scale reliability. Fisher's exact probability test (multiple comparisons were corrected using the Benjamini and Hochberg method) was used to compare the proportions of qualitative variables among the groups. For the comparison of quantitative variables among groups, the Kruskal-Wallis test for nonparametric variables (Dunn-Bonferroni method for multiple comparisons), analysis of variance (ANOVA) for parametric variables (Bonferroni method for multiple comparisons), and *t*-test were used. An exploratory factor analysis (unweighted least squares) was conducted to determine savant tendency. Spearman's rank correlation coefficients were calculated to examine the relationships among quantitative and ordinal variables.

As Exploratory analysis, for all participants in the KS, sexual minority, and control 1st, a stepwise logistic regression analysis was conducted to examine whether each variable predicted synesthesia and a multiple regression analysis using the stepwise method was conducted to examine whether each variable predicted the savant tendency and the total GSQ score, respectively.

As Reanalysis, for all participants in the KS, sexual minority, and control 2nd, additional exploratory analyses were performed. To examine whether GSQ or gender dysphoric state better explained savant tendency or synesthesia, multiple regression analysis with the forced entry method with savant tendency as the dependent variable and logistic regression analysis with the forced entry method with synesthesia as the dependent variable were conducted.

The above analyses were conducted using SPSS software [IBM SPSS Statistics 28.0.1.0 (142)]. R (4.1.3) was used for data plotting, Fisher's exact probability test administration, and the calculation of Cohen's *d* values. The significance level was set at *α* = .05 without adjusting for multiple testing across analyses.

## 3 Results

### 3.1 Exploratory analysis (KS, sexual minority, and control 1st)

The numbers of participants for analyses were 22 in the KS group (*Mean* age = 42.41 years, *SD* = 8.11), 66 in the sexual minority group (*Mean* age = 34.35 years, *SD* = 12.81), and 36 in the control 1st group (*Mean* age = 42.67 years, *SD* = 7.64).

The karyotype of the KS group was 47, XXY (*n* = 22). The diagnosis histories of the syndrome were as follows: 13 patients were diagnosed with infertility (one of them was told of the possibility of KS during an infertility check but left untreated for approximately 10 years, and later discovered when he visited a urologist for another problem), and nine patients were because of reasons other than infertility (discovered by chromosome examination during the diagnosis of GID, *n* = 2; suspected during a visit to a urologist, *n* = 2; suspected by themselves based on other symptoms or physical characteristics, *n* = 4; a sudden announcement by a doctor in adulthood, although it was discovered during a hospital visit for another condition in childhood, *n* = 1).

The number of individuals who experienced hormone administration/treatment was 17 in the KS group (androgen *n* = 11, estrogen *n* = 3, both hormones *n* = 3), 31 in the sexual minority group (androgen *n* = 1, estrogen *n* = 29, antiandrogen *n* = 1), and 0 in the control 1st group.

#### 3.1.1 Demographic data

Supplementary S2 shows the percentages or representative values of each variable in the demographic data and the results of the group comparisons.

Table 1 shows the percentage and frequency of each developmental disorder, as well as the results of the group comparisons. The percentage of respondents diagnosed with ASD was 15.2% in the sexual minority group, which was significantly higher than that in the control 1st group.

**Table 1:** Percentage and frequency of diagnosis of developmental disorders and results of group comparisons.

Diagnosis	KS (*n* = 22)	Sexual minority (*n* = 66)	Control 1st (*n* = 36)	*p*-value	Multiple comparisons
ASD	4.5 (1)	15.2 (10)	0 (0)	.017	K = S, K = C, S > C
ADHD	13.6 (3)	16.7 (11)	2.8 (1)	.097	
Dyslexia	0 (0)	1.5 (1)	0 (0)	>.999	
Dysgraphia	4.5 (1)	1.5 (1)	0 (0)	.407	
Dyscalculia	4.5 (1)	1.5 (1)	0 (0)	.407	

Note: Each cell represents % (*n*) in the group. *P*-values represent the findings of the Fisher's exact probability test. In multiple comparisons, K represents the KS group, S represents the sexual minority group, and C represents the control 1st group.

Histories of other diseases and psychiatric symptoms are described in Supplementary S3, S4, respectively.

#### 3.1.2 Sexual aspects

Table 2 shows the percentages or representative values of each variable in the sexual aspects, as well as the results of the group comparisons.

**Table 2:** Percentage or representative value of each variable in the sexual aspects and results of group comparisons.

Variable	KS (*n* = 22)	Sexual minority (*n* = 66)	Control 1st (*n* = 36)	*p*-value	Multiple comparisons^c^
Gender dysphoric state^a^ (yes)	31.8 (7)	68.2 (45)	0 (0)	<.001	K < S, K > C, S > C
Diagnosis of GID/GD^a^	9.1 (2)	39.4 (26)	0 (0)	<.001	K < S, K = C, S > C
Kinsey score^b^	1.00 ± 0.38	5.17 ± 1.83	1.00 ± 0.00	<.001	K < S, K = C, S > C
Masculinity of the play^b^	.13 ± 0.28	−.33 ± 0.33	.71 ± 0.29	<.001	K > S, K < C, S < C
Cross-dressing^b^	1.00 ± 1.0	4.00 ± 2.0	1.00 ± 0.0	<.001	K < S, K = C, S > C
Male friends in childhood^b^	4.00 ± 1.5	3.00 ± 1.5	2.00 ± 2.0	.133	

Note: Kinsey score consisted of sexual minority (*n* = 64) and control 1st (*n* = 35). Masculinity of the play consisted of control 1st (*n* = 34).

^a^ Each cell represents % (*n*) in each group, with the *p*-value determined from the Fisher's exact probability test.

^b^ Each cell represents median ± quartile deviation, with the *p*-value determined from the Kruskal-Wallis test.

^c^ K in each cell represents the KS group, S represents the sexual minority group, and C represents the control 1st group.

With respect to gender dysphoric state, we categorized the responses of other (free description) as “other (nonbinary)”, if the description indicated being nonbinary or expressed discomfort with being male, and “yes (trans)”, if the description indicated apparent GD, such as GID. In the following analyses, the data of “other (nonbinary)” and “yes (trans)” were combined to “yes” in the “gender dysphoric state (yes/no)” category. The order of frequency of the gender dysphoric state was as follows: sexual minority group (most frequent), KS group, and control 1st group (least frequent). The distribution of “other (nonbinary)” and “yes (trans)” is shown in Supplementary S5.

In the KS group, gender dysphoric states were expected to differ depending on the reasons for the KS diagnosis (for infertility or not); thus, we present the divided analyses of gender dysphoric state and other phenotypes based on the diagnosis history in Supplementary S6.

Kinsey scores were significantly higher in the sexual minority group than in the KS and the control 1st groups. The degree of masculinity of the play was highest in the control 1st group, followed by the KS and sexual minority groups.

#### 3.1.3 Synesthesia

Those who were considered to have (or had had) synesthesia were *n* = 2 (9.1%) in the KS group, *n* = 12 (18.2%) in the sexual minority group, and *n* = 1 (2.8%) in the control 1st group. Group differences were not significant, based on Fisher's exact test (*p* = .065). The details of each participant's synesthetic state are presented in Supplementary S7.

#### 3.1.4 Savant tendency

To examine whether the group of savant tendency items could be considered a one-dimensional scale, factor analysis was conducted using unweighted least squares with a one-factor solution. The sum of squares of the loadings after extraction was 23.68% of the variance, with low commonality (.08) and factor loading (.28) on the item “I have memories of events prior to the age of 2”. The item “I tend to experience and be influenced by other people's emotions” did not have a high factor loading (.36), nor is it a trait that has been referred to in savant syndrome (32, 37). Rather, the content of the items is close to that of “empathy” toward others (2), which is considered weak in individuals with high ASD traits. Therefore, we omitted these two items and reanalyzed them using exploratory factor analysis. The sum of the squares of the loadings after extraction accounted for 25.33% of the variance. The factor loadings for each item are listed in Table 3. Reliability (17 items in total) was *α* = .85. The total score of these 17 items was used as the savant tendency score in Exploratory analysis.

**Table 3:** Factor loadings (savant tendency) in study I.

Item	Factor loadings
I am good at learning new languages.	.50
I am able to recall in detail a scene watched only once.	.62
I can remember details of what I heard only once by ear, such as lines from a movie or drama or a person's story.	.48
I am good at memorization (e.g., train schedules, phone numbers, addresses, past weather…)	.61
I am good at remembering maps and directions.	.54
I am good at finding where a place is on a map after looking at its picture or drawing.	.63
I am good at making three-dimensional objects such as Lego, 3D video games (e.g., Minecraft), sculpting, DIY, etc.	.55
I am good at solving jigsaw puzzles.	.59
I can play/have experienced playing an instrument without learning it first.	.43
I can play music on an instrument after hearing it only once.	.44
I can recognize the musical scale (do-re-mi…) just by listening to a sound.	.42
I am good at drawing.	.35
I can quickly tell what day of the week a certain date (what year, what month, what day of the week) corresponds to.	.40
I am good at mental arithmetic.	.45
I can tell right away if a number is prime by looking at it.	.57
I am a geek of some kind.	.42
I know how animals feel.	.43

**Figure 2:**
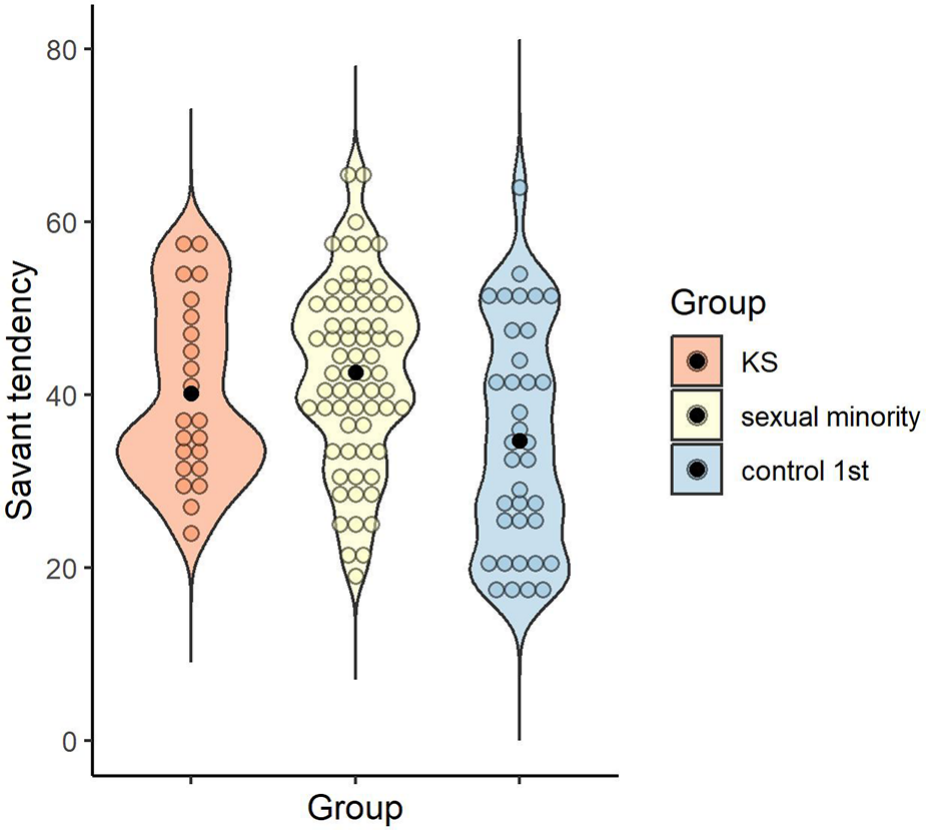
Distribution of savant tendency score in each group. Black dots indicate the mean for each group.

The mean and standard deviation of the savant tendency for each group were calculated: *M* = 40.14 (*SD* = 10.32) for the KS group, *M* = 42.58 (*SD* = 10.71) for the sexual minority group, and *M* = 34.69 (*SD* = 12.92) for the control 1st group. A one-way ANOVA was conducted to examine whether the savant tendency differed by group, and the main effect of group was significant [*F* (2, 121) = 5.64, *p* = .005, *η_p_^2^* = .09, 1 - β = .85]. Multiple comparisons revealed that the sexual minority group scored higher than the control 1st group (*p* = .003, *d* = 0.68), with no significant difference between the KS and sexual minority groups (*p* > .999, *d* = 0.23), or between the KS and control 1st groups (*p* = .235, *d* = 0.45) (Figure 2).

#### 3.1.5 GSQ (sensory hypersensitivity/hyposensitivity)

The reliability of the total GSQ score was *α* = .95. The mean and standard deviation of the GSQ total scores for each group were calculated: *M* = 48.68 (*SD* = 27.13) for the KS group, *M* = 53.38 (*SD* = 22.59) for the sexual minority group, and *M* = 25.14 (*SD* = 25.18) for the control 1st group.

**Figure 3:**
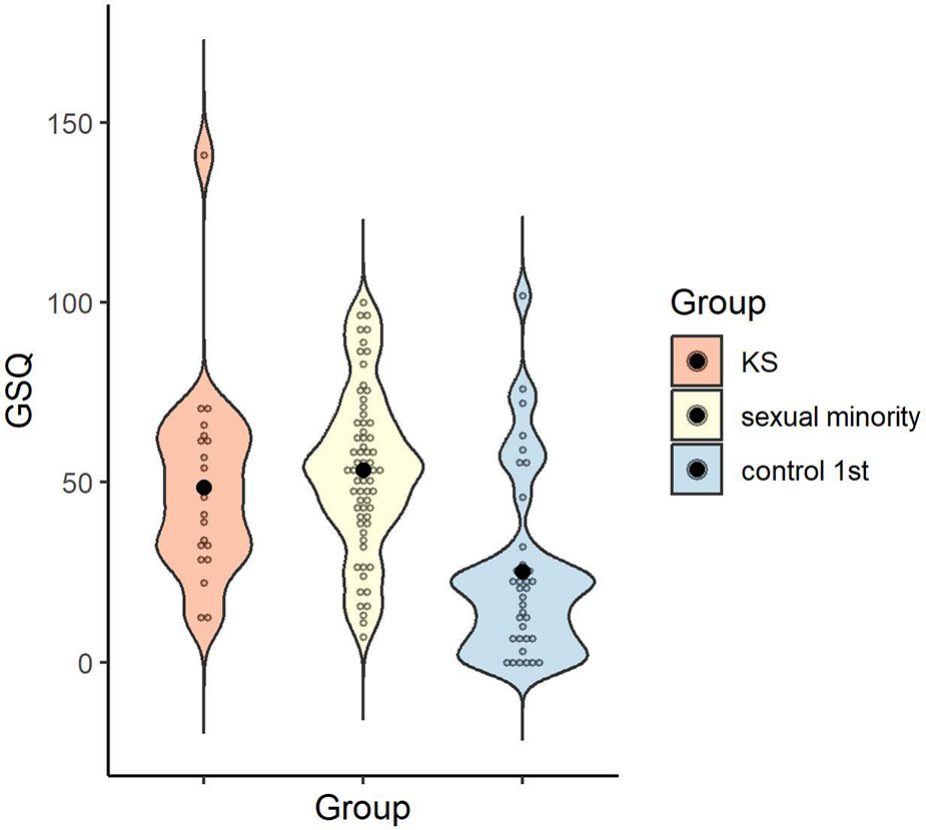
Distribution of GSQ total score in each group. Black dots indicate the mean for each group. GSQ, Glasgow sensory questionnaire.

A one-way ANOVA was conducted to examine whether the GSQ total scores differed by group, and the main effect of group was significant [*F* (2, 121) = 16.30, *p* < .001, *η_p_^2^* = .21, 1 - β > .99]. Multiple comparisons revealed higher sensory hypersensitivity/hyposensitivity in the KS and sexual minority groups than in the control 1st group (*p* = .001, *d* = 0.91; *p* < .001, *d* = 1.20), with no significant difference between the KS and sexual minority groups (*p* > .999, *d* = .20) (Figure 3).

#### 3.1.6 Correlation

Spearman's rank correlation coefficients were calculated for all the participants (*n* = 124) for each quantitative and ordinal variable (Table 4). The item “I tend to experience and be influenced by other people's emotions”, which was excluded from the savant tendency score, was included in the correlation analysis under the variable name of “*empathic sensitivity*” as it is a concept similar to empathy (2) and was expected to be related to the other variables.

**Table 4:** Correlation coefficient between each variable.

Spearman's *ρ*	a	b	c	d	e	f	g	h
Savant tendency: a								
Empathic sensitivity: b	.30^b^							
Kinsey score: c	.25^b^	.29^b^						
Masculinity of the play: d	−.25^b^	−.33^b^	−.51^b^					
Cross-dressing: e	.21^a^	.28^b^	.37^b^	−.40^b^				
Male friends in childhood: f	−.07	−.06	.01	.06	−.10			
Age: g	−.19^a^	−.22^a^	−.41^b^	.13	.01	−.03		
Educational background: h	−.13	−.04	−.04	.10	.04	.00	−.07	
GSQ total score: i	.22^a^	.36^b^	.28^b^	−.36^b^	.50^b^	−.04	−.13	−.10

Note: *n* = 121 for correlation with Kinsey score, *n* = 122 for correlation with masculinity of the play. The “educational background: h” was an ordinal variable with “middle school graduate = 1∼ doctoral degree 5 = 7”.

^a^ Significant at the 5% level.

^b^ Significant at the 1% level.

#### 3.1.7 Predicting synesthesia: logistic regression analysis

To examine what variables predicted synesthesia (yes/no), logistic regression analysis with a variable increase stepwise method was conducted based on likelihood ratio with the dependent variable as “synesthesia (yes/no)” and the candidates of predictor variables as “diagnosis of KS (yes/no)”, “hormone treatment (yes/no)”, “savant tendency”, “empathic sensitivity”, “gender dysphoric state (yes/no)”, “Kinsey score”, “masculinity of the play”, “male friends in childhood”, “age”, “handedness (right-handed/non-right-handed)”, “educational background”, “mental illness (yes/no)”, “diagnosis of developmental disorder (yes/no)”, “GSQ” (unlike the OSF, diagnosis of GD/GID and cross-dressing were not included to restrain multicollinearity), and a model was obtained in two steps. Step 1 yielded a significant model predicted by “GSQ” [Wald = 7.59, *p* = .006, Exp (β) = 1.03, 95% CI (1.01–1.06), Nagelkerke R^2^ = .14]. Predictor variables in Step 2 did not reach significance, as follows: “GSQ” [Wald = 3.54, *p* = .060, Exp (β) = 1.02, 95% CI (1.00–1.05)] and “gender dysphoric state” [Wald = 3.58, *p* = .058, Exp (β) = 3.90, 95% CI (0.95–15.98)] (Nagelkerke R^2^ = .20).

#### 3.1.8 Predicting savant tendency: multiple regression analysis

To examine what variables predicted the savant tendency, a multiple regression analysis was performed using a stepwise method with the dependent variable as “savant tendency” and the candidates of predictor variables as “diagnosis of KS (yes/no)”, “hormone treatment (yes/no)”, “synesthesia (yes/no)”, “empathic sensitivity”, “gender dysphoric state (yes/no)”, “Kinsey score”, “masculinity of the play”, “male friends in childhood”, “age”, “handedness (right-handed/non-right-handed)”, “educational background”, “mental illness (yes/no)”, “diagnosis of developmental disorder (yes/no)”, “GSQ” (unlike the OSF, diagnosis of GD/GID and cross-dressing were not included to restrain multicollinearity). The results yielded models predicted by “empathic sensitivity” (β = .27, *p* = .003), “handedness” (β = .19, *p* = .026), and “gender dysphoric state” (β = .19, *p* = .032) (adjusted R^2^ = .17, *p* < .001) (Supplementary S11).

#### 3.1.9 Predicting GSQ: multiple regression analysis

To examine what variables predicted the GSQ, multiple regression analysis was conducted using the stepwise method with the dependent variable as “GSQ” and the candidates of predictor variables as “diagnosis of KS (yes/no)”, “hormone treatment (yes/no)”, “synesthesia (yes/no)”, “savant tendency”, “empathic sensitivity”, “gender dysphoric state (yes/no)”, “Kinsey score”, “masculinity of the play”, “male friends in childhood”, “age”, “handedness (right-handed/non-right-handed)”, “educational background”, “mental illness (yes/no)”, “diagnosis of developmental disorder (yes/no)” (unlike the OSF, diagnosis of GD/GID and cross-dressing were not included to restrain multicollinearity). The results yielded models predicted by “gender dysphoric state” (β = .29, *p* = .001), “diagnosis of developmental disorder” (β = .23, *p* = .008), and “empathic sensitivity” (β = .20, *p* = .015) (adjusted R^2^ = .29, *p* < .001) (Supplementary S12).

### 3.2 Reanalysis (KS, sexual minority, and control 2nd)

The participants in the analysis comprised the control 2nd group, *n* = 583 (*Mean* age = 42.02 years, *SD* = 7.67). The KS and sexual minority groups were described in Exploratory analysis (see 3.1). In the control 2nd group, *n* = 39 (6.7%) were diagnosed with mental illness and *n* = 11 (1.9%) were diagnosed with developmental disorders. In the control 2nd group, we did not ask whether the participants experienced gender dysphoric state, but we assumed that they did not because they responded to the recruitment for male participants and chose not to opt for the category of LGBTs in the survey.

#### 3.2.1 Comparison of three groups

We examined whether the results of the three-group comparisons (KS vs. sexual minority vs. control 1st) conducted in Exploratory analysis (see 3.1.3–3.1.5) could be replicated.

##### 3.2.1.1 Synesthesia

The number of participants considered to have (or had had) synesthesia was 10 (1.7%) in the control 2nd group (KS group: *n* = 2 (9.1%); sexual minority group: *n* = 12 (18.2%); see 3.1.3). A description of synesthesia in the control 2nd group is provided in Supplementary S13.

Group differences were considered significant based on Fisher's exact probability test (*p* < .001). Multiple comparisons revealed a difference between the sexual minority and control 2nd groups (*p* < .001), but not between the KS and sexual minority groups or between the KS and control 2nd groups (*p* = .503 and *p* = .134, respectively).

##### 3.2.1.2 Savant tendency

Including all samples (KS, sexual minority, and control 2nd groups), factor analysis was conducted using unweighted least squares with a one-factor solution to examine whether the group of savant tendency items could be considered on a single-dimensional scale. The sum of the squares of the loadings after extraction was 26.44% of the variance, with no items having low commonality (.1 or less). All factor loadings were greater than.35. We reanalyzed them with exploratory factor analysis excluding the item “I tend to experience and be influenced by other people's emotions”, as was done in 3.1.4. The sum of the squares of the loadings after extraction constituted 27.06% of the variance. The item “I am a geek of some kind” did not have a high factor loading, as it was less than .35 (.348). Therefore, we excluded them and reanalyzed using factor analysis. The sum of the squares of the loadings after extraction accounted for 27.94% of the variance. The factor loadings for each item are listed in Table 5. Reliability (17 items in total) was *α* = .86. The total score for these 17 items was used as the savant tendency score in Reanalysis.

**Table 5:** Factor loadings (savant tendency) in study II.

Item	Factor loadings
I am good at learning new languages.	.53
I am able to recall in detail a scene watched only once.	.59
I have memories of events prior to the age of 2.	.42
I can remember details of what I heard only once by ear, such as lines from a movie or drama or a person's story.	.59
I am good at memorization (e.g., train schedules, phone numbers, addresses, past weather…).	.62
I am good at remembering maps and directions.	.47
I am good at finding where a place is on a map after looking at its picture or drawing.	.60
I am good at making three-dimensional objects such as Lego, 3D video games (e.g., Minecraft), sculpting, DIY, etc.	.53
I am good at solving jigsaw puzzles.	.53
I can play/have experienced playing an instrument without learning it first.	.52
I can play music on an instrument after hearing it only once.	.54
I can recognize the musical scale (do-re-mi…) just by listening to a sound.	.51
I am good at drawing.	.38
I can quickly tell what day of the week a certain date (what year, what month, what day of the week) corresponds to.	.50
I am good at mental arithmetic.	.55
I can tell right away if a number is prime by looking at it.	.59
I know how animals feel.	.46

**Figure 4:**
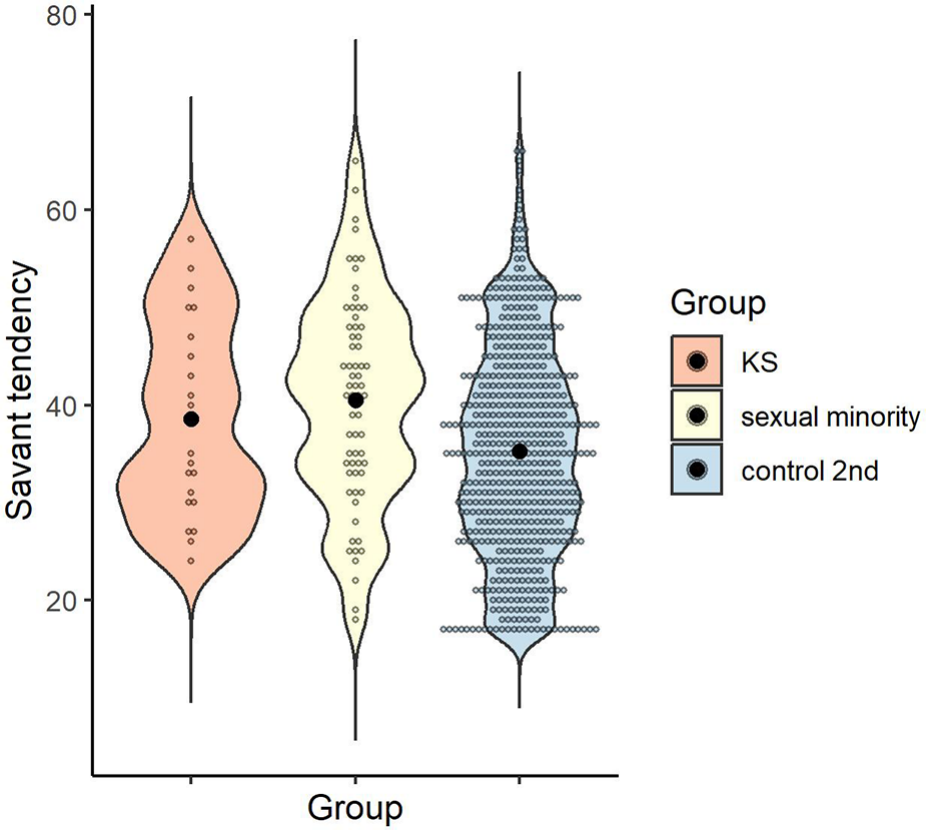
Distribution of savant tendency score in each group. Black dots indicate the mean for each group.

The mean and standard deviation for each group were calculated for savant tendency, as follows: *M* = 38.55 (*SD* = 9.99) for the KS group, *M* = 40.47 (*SD* = 10.62) for the sexual minority group, and *M* = 35.25 (*SD* = 10.70) for control 2nd group. A one-way ANOVA was conducted to examine whether savant tendency differed by group, and the main effect of group was significant [*F* (2, 668) = 7.80, *p* < .001, *η_p_*^2^ = .02, 1-β = .95]. Multiple comparisons revealed that the sexual minority group scored higher than the control 2nd group (*p* < .001, *d* = 0.49), with no significant difference between the KS and sexual minority groups or between the KS and control 2nd groups (*p* > .999, *d* = 0.18; *p* = .468, *d* = 0.31) (Figure 4).

##### 3.2.1.3 GSQ

**Figure 5:**
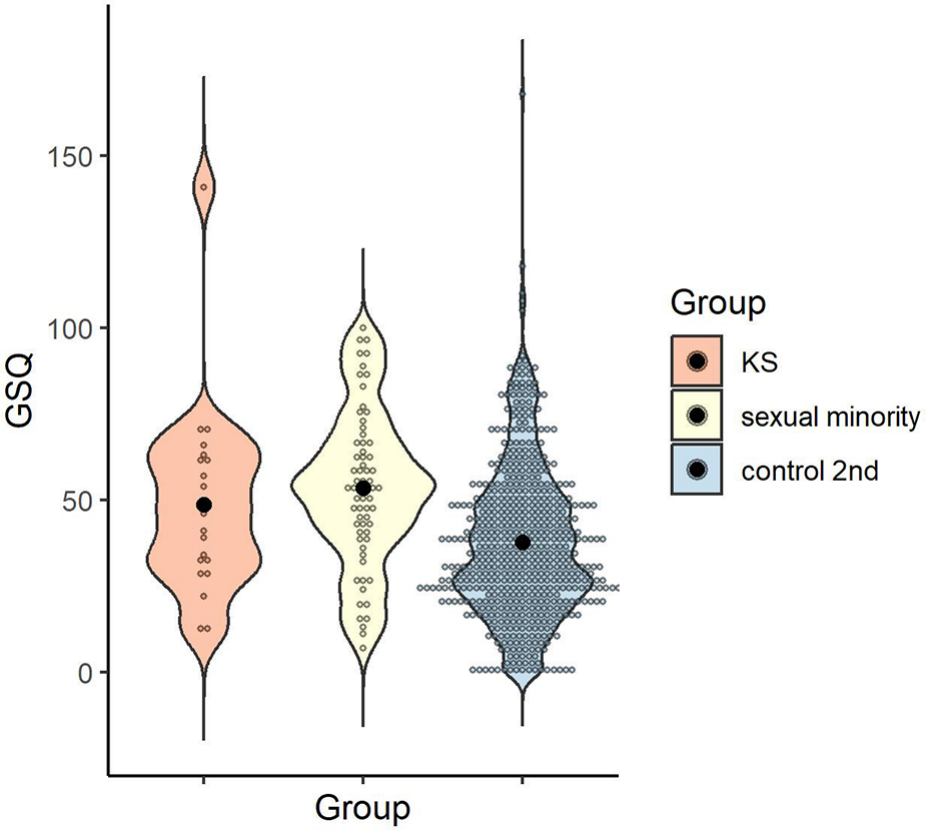
Distribution of GSQ total score in each group. Black dots indicate the mean for each group. GSQ, Glasgow sensory questionnaire.

The reliability of the total GSQ score was *α* = .95. The mean and standard deviation of the control 2nd group in the GSQ total scores were calculated, as follows: *M* = 37.79, *SD* = 22.33 (KS group: *M* = 48.68, *SD* = 27.13; sexual minority group: *M* = 53.38, *SD* = 22.59; see 3.1.5). One-way ANOVA was conducted to examine whether GSQ total scores differed by group, and the main effect of group was significant [*F* (2, 668) = 16.06, *p* < .001, *η_p_*^2^ = .05, 1-β > .99]. Multiple comparisons revealed that the sexual minority group scored higher than the control 2nd group (*p* < .001, *d* = 0.70), with no significant difference between the KS and sexual minority groups or between the KS and control 2nd groups (*p* > .999, *d* = 0.20; *p* = .079, *d* = 0.48) (Figure 5).

#### 3.2.2 Association with gender dysphoric state

To examine whether gender dysphoric state was related to sensory and cognitive characteristics, as suggested in Exploratory analysis (see 3.1), we examined whether sensory hypersensitivity/hyposensitivity (GSQ scores), savant tendency, and synesthesia differed depending on gender dysphoric state. The hypotheses were as follows: (1) “individuals with gender dysphoric state are more likely to be synesthetes than are those without”, (2) “individuals with gender dysphoric state have a higher savant tendency than those of individuals without”, (3) “individuals with gender dysphoric state have a higher tendency toward hypersensitivity/hyposensitivity than that of individuals without”.

##### 3.2.2.1 Synesthesia

Fisher's exact probability test was conducted to examine the association between synesthesia (yes/no) and gender dysphoric state (yes/no), and the results were significant (*p* < .001) (Table 6). The odds ratio was 15.02 (95% CI: 5.78–39.22).

**Table 6:** Percentage in synesthesia and gender dysphoric state.

		Gender dysphoric state	
		No (*n* = 619)	Yes (*n* = 52)
Synesthesia	No (*n* = 647)	98.1 (607)	76.9 (40)
	Yes (*n* = 24)	1.9 (12)	23.1 (12)

Note: Each cell represents percentage, and the numbers in parentheses represent frequencies.

##### 3.2.2.2 Savant tendency

**Figure 6:**
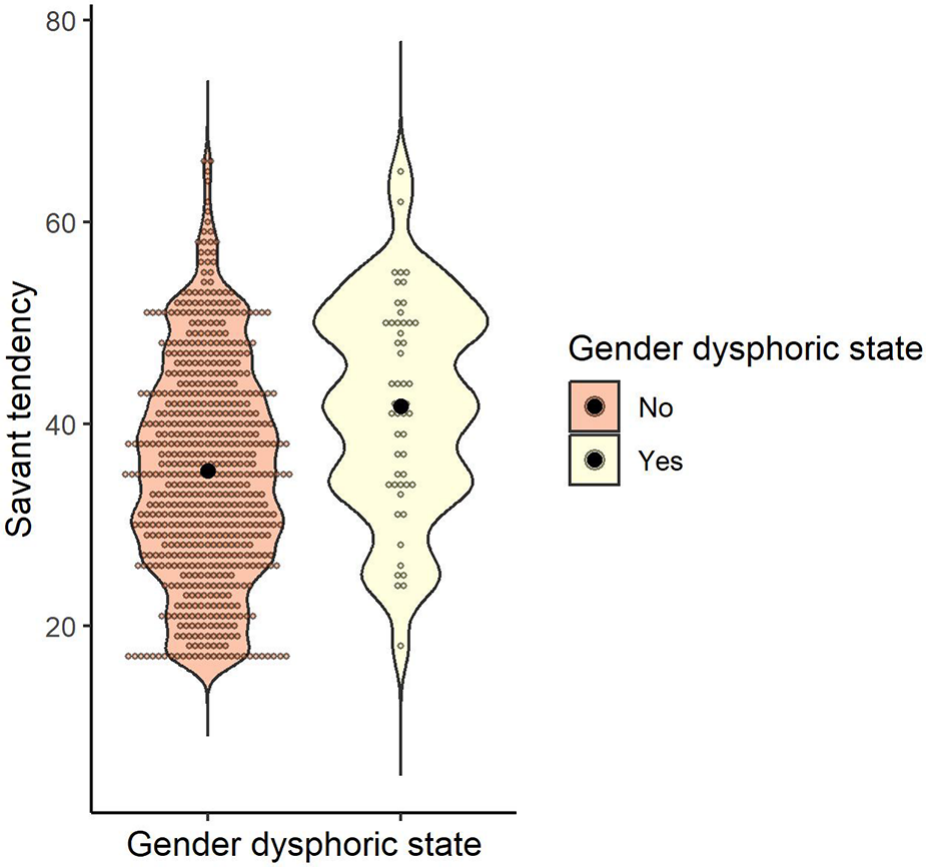
Distribution of savant tendency score by gender dysphoric state. Black dots indicate the mean for each group.

The mean and standard deviation of the savant tendency were calculated for gender dysphoric state (yes/no): *M* = 35.38 (*SD* = 10.67) for those who answered no (*n* = 619) and *M* = 41.73 (*SD* = 10.49) for those who answered yes (*n* = 52). To examine whether the savant tendency differed according to the gender dysphoric state, a *t*-test was conducted, and the result was significant, indicating that the presence of a gender dysphoric state was associated with a higher savant tendency [*t* (669) = 4.13, *p* < .001, *d* = 0.60] (Figure 6).

##### 3.2.2.3 GSQ

**Figure 7:**
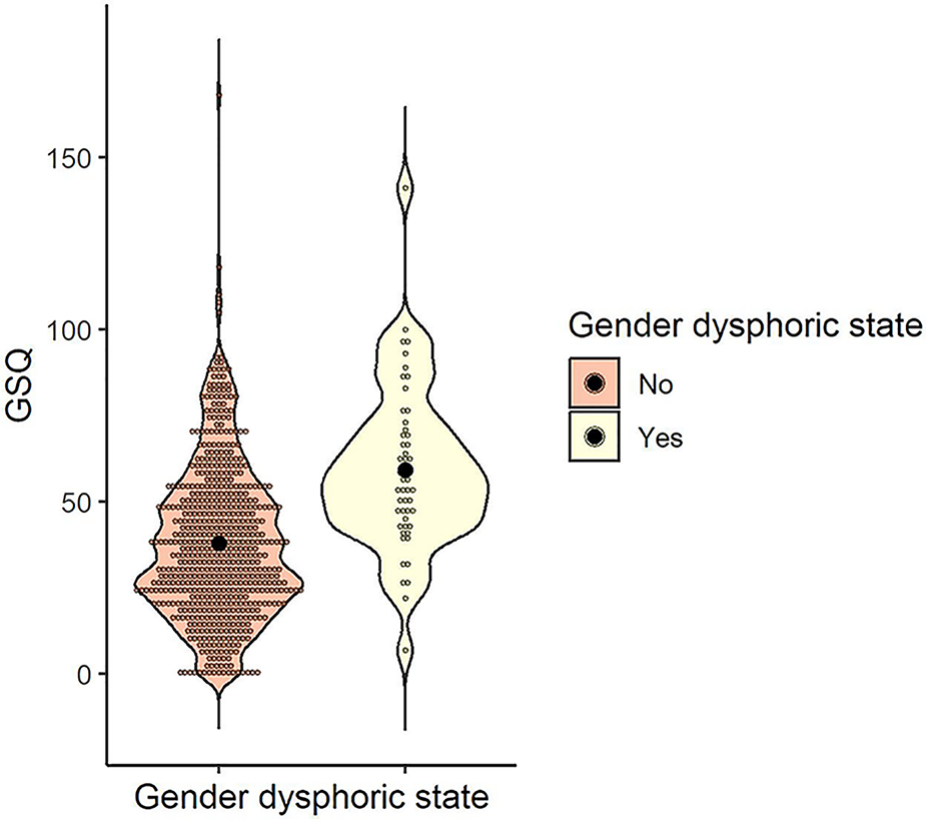
Distribution of GSQ total score by gender dysphoric state. Black dots indicate the mean for each group. GSQ, Glasgow sensory questionnaire.

The mean and standard deviation of the GSQ total scores for the gender dysphoric state (yes/no) were calculated as follows: *M* = 38.04 (*SD* = 22.25) for those who answered no (*n* = 619) and *M* = 59.21 (*SD* = 23.26) for those who answered yes (*n* = 52). To examine whether the GSQ differed by gender dysphoric state, a *t*-test was conducted and was significant, indicating that the presence of a gender dysphoric state was associated with a higher GSQ score [*t* (669) = 6.57, *p* < .001, *d* = 0.95] (Figure 7).

#### 3.2.3 Correlation

**Figure 8:**
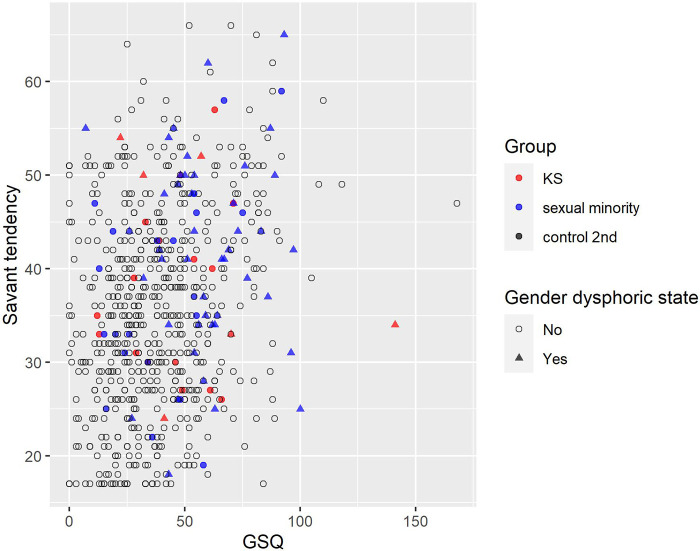
Association of savant tendency score and GSQ total score by gender dysphoric state. Red, blue, and black indicate KS, sexual minority, and control 2nd, respectively. 〇 indicates no (not having gender dysphoric state) and △ indicates yes (having gender dysphoric state). GSQ, Glasgow sensory questionnaire; KS, Klinefelter syndrome.

As a result of Spearman's rank correlation coefficient for all the participants (*n* = 671), weak positive correlations were found in savant tendency and GSQ (*ρ* = .23). Scatter plots between savant tendency and GSQ are shown, along with the presence of a gender dysphoric state (Figure 8). In Supplementary S14, scatter plots between savant tendency and GSQ are shown, along with the presence of a developmental disorder. Many participants with a gender dysphoric state or a diagnosis of developmental disorder had high scores on both savant tendency and GSQ, but some high scorers did not have either. In addition, correlation coefficients other than above are listed in the Supplementary S15.

#### 3.2.4 Additional exploratory analysis

##### 3.2.4.1 Predicting synesthesia: logistic regression analysis

To examine whether gender dysphoric state or GSQ was more predictive of synesthesia (yes/no), we conducted a logistic regression analysis using the forced-choice method with “synesthesia (yes/no)” as the dependent variable and gender dysphoric state and GSQ as the predictor variables. The results yielded a model predicted by “gender dysphoric state” [Wald = 24.54, *p* < .001, Exp (β) = 9.80, 95% CI (3.97–24.18)] and “GSQ” [Wald = 9.02, *p* = .003, Exp (β) = 1.03, 95% CI (1.01–1.04)] (Nagelkerke R^2^ = .22). In addition, when “diagnosis of developmental disorder” was included as a predictor variable, regression coefficients were not significant (Supplementary S17).

##### 3.2.4.2 Predicting savant tendency: multiple regression analysis

To examine whether gender dysphoric state or GSQ was more predictive of savant tendency, we conducted a multiple regression analysis using the forced-choice method with “savant tendency” as the dependent variable and gender dysphoric state and GSQ as the predictor variables. As a result, we obtained a model in which “gender dysphoric state” (β = .10, *p* = .010) and “GSQ” (β = .24, *p* < .001) were predictive (adjusted R^2^ = .07, *p* < .001) (Supplementary S16). In addition, when “diagnosis of developmental disorder” was included as a predictor variable, regression coefficients were not significant (Supplementary S17).

## 4 Discussion

In the present study, to examine whether ASD traits are found in samples with assumed androgen deprivation, we conducted an exploratory study of sensory hypersensitivity/hyposensitivity, savant tendency, synesthesia, and sexual aspects among individuals with KS, sexual minorities assigned male at birth, and control males (control 1st and control 2nd group).

As a result, the KS group showed a greater tendency toward sensory hypersensitivity/hyposensitivity compared to the control group in Exploratory analysis. The sexual minority group was more likely to exhibit synesthesia in Reanalysis. Both Exploratory analysis and Reanalysis showed that the sexual minority group tended to have a higher savant tendency and sensory hypersensitivity/hyposensitivity than the control group. Additionally, the item “I tend to experience and be influenced by other people's emotions (tagged as *empathic sensitivity*)”, which was excluded from the savant tendency, was related to various variables such as savant tendency, and sensory hypersensitivity/hyposensitivity. Moreover, the gender dysphoric state is associated with phenotypes commonly observed in individuals with ASD, such as synesthesia, savant tendency, and sensory hypersensitivity/hyposensitivity.

The results of the present study suggest that androgynous features (reduced sex steroid action during early development) in males at birth are partially related to the phenotypes observed in individuals with ASD, particularly, the possibility of a common physiological background between GD and synesthesia, savant tendency, and sensory hypersensitivity/hyposensitivity.

### 4.1 Synesthesia

The sexual minority group was more likely to exhibit synesthesia (see 3.2.1.1), although it did not reach significance in Exploratory analysis (see 3.1.3). It has been reported that at least 10% of synesthetes are homosexuals (38) and 33% of MTF transgenders are synesthetes (39), suggesting that synesthetes appear at higher rates in sexual minorities, as in the present study.

Moreover, those with gender dysphoric state were more likely to have synesthesia (see 3.2.2.1). Synesthesia was predicted by the scores of GSQ in Exploratory analysis (see 3.1.7) and by gender dysphoric state and GSQ in additional exploratory analysis (see 3.2.4.1). These are consistent with previous reports of higher sensory hypersensitivity/hyposensitivity in synesthetes (28) and higher prevalence of synesthetes in MTF transgenders (39). MTF transgenders have increased cortical areas (e.g., fusiform gyrus and inferior temporal gyrus) that are also associated with grapheme-color synesthesia, suggesting it may be related to the susceptibility of co-occurrence of both (8).

### 4.2 Savant tendency

Factor analysis yielded a highly reliable 17-item scale. With respect to conceptual validity (content validity), savants are difficult to examine because there are no objective criteria, and the definition is not clear; however, the items were created based on the contents mentioned in studies by Hughes et al. (32) and Treffert (37). This scale also makes it possible to quantitatively measure characteristics related to abilities observed in savants, including the general population, rather than using a dualistic interpretation of savants or non-savants. A significant positive correlation with the GSQ (see Table 4; 3.2.3, 3.2.4.2) was found for construct validity, and given the association between savant tendency and sensory hypersensitivity/hyposensitivity (32), construct validity is somewhat warranted.

The sexual minority group tended to have a higher savant tendency (see 3.1.4; 3.2.1.2). Specifically, those with gender dysphoric state were more likely to have a higher savant tendency (see 3.2.2.2). Furthermore, the savant tendency was predicted by empathic sensitivity, handedness, and gender dysphoric state in Exploratory analysis (see 3.1.8) and by gender dysphoric state and GSQ in additional exploratory analysis (see 3.2.4.2).

Given the associations between ASD and savant abilities (37), and sensory hypersensitivity/hyposensitivity (36), a common neurophysiological basis may facilitate the development of similar phenotypes between sexual minorities (especially, gender dysphoria) and ASD. Including savant ability, phenotypes observed in ASD may be associated with the development of heterogeneity, such as atypical sensory sensitivity, beginning with sex steroid effects during early development (8). Although the arguments connecting gender dysphoria to ASD susceptibility have been made regarding the causal processes of social impairments and other disabilities related to ASD, it is groundbreaking to show that these arguments can also be applied to cognitive strength such as savant tendency. Moreover, the present study suggests that savant ability may be related to sensory hypersensitivity/hyposensitivity. The results are in line with some theories, such as the EPF model (30) and Baron-Cohen's theory (atypical senses promote attention to detail, which develops into systemizing and leads to savant ability) (31).

Furthermore, the present study found a positive association between empathic sensitivity and savant tendency. The item “empathic sensitivity” overlaps with the concept of the highly sensitive person (HSP) (40). Although empathy skill is perturbed in individuals with ASD (2) and is negatively related to the effects of early testosterone (41), empathic sensitivity was positively associated with sensory hypersensitivity/hyposensitivity that is commonly observed in ASD in the present study. It has been noted that although cognitive empathy, such as the theory of mind, which requires conscious processing of emotions, is impaired in individuals with ASD, emotional empathy, such as sharing sensations with others, is similar or may even be stronger in individuals with ASD than in those with typical development (42). This can be said to represent the blurring of the boundaries between the self and others in individuals with ASD (42). It is also said that emotional overexcitement is a characteristic of giftedness (43). The results of the present study are consistent with these arguments. However, because “empathic sensitivity” is a single-item measure, it should be examined in more detail in future studies.

Regarding handedness, the result suggested a higher savant tendency among non-right-handed individuals. In a meta-analysis, non-right-handedness was more prevalent in individuals with ASD than in those with typical development, and non-right-handedness was considered to have stronger right-brain hemisphere dominance (44). Savant abilities are also thought to be associated with the right hemispheric function (37), and the result of the present study is consistent with these findings.

### 4.3 GSQ (sensory hypersensitivity/hyposensitivity)

The sexual minority group tended to have a higher GSQ score (see 3.1.5; 3.2.1.3). Those with gender dysphoric state were more likely to have a higher GSQ score (see 3.2.2.2). Furthermore, The GSQ was predicted by gender dysphoric state, diagnosis of developmental disorder, and empathic sensitivity in Exploratory analysis (see 3.1.9).

With respect to gender dysphoric state, the result was consistent with that of a study using the Sensory Perception Quotient-10, which reported that transgender and gender-diverse individuals’ scores were higher than those of cisgender individuals (45). The results of the present study also suggested that gender identity might be more closely related to atypical sensory perception than to a KS diagnosis or sexual orientation.

Regarding developmental disorders, the result suggested that sensory hypersensitivity/hyposensitivity was higher in individuals with developmental disorders. This result is consistent with those of various developmental disorders, including ASD, which tend to present with sensory symptoms (46).

The result of empathic sensitivity suggested that sensory hypersensitivity/hyposensitivity was higher in those with higher levels of empathic sensitivity. The result was consistent with the abovementioned discussion on emotional empathy and emotional overexcitation in individuals with ASD and in gifted individuals.

### 4.4 Differences in results between KS and sexual minorities

With respect to the difference in results between individuals with KS and sexual minorities in the present study, individuals with KS only showed higher sensory hypersensitivity/hyposensitivity than that exhibited by the controls in Exploratory analysis (see 3.1), whereas sexual minorities showed a higher number of synesthetes and a higher savant tendency and sensory hypersensitivity/hyposensitivity than those exhibited by the controls (see 3.2). On comparing individuals with KS to sexual minorities, the lower association with phenotypes observed in ASD may reflect the wide variations in sensory and cognitive characteristics depending on the reasons for the KS diagnosis (for infertility or not) in the KS population. Although statistical tests were not conducted owing to the small sample size, KS individuals diagnosed with conditions other than infertility tended to have more GD, a diagnosis of developmental disorders, and higher mean values for savant tendency and sensory hypersensitivity/hyposensitivity compared with the same parameters observed in KS individuals diagnosed with infertility (Supplementary S6). In the present study, 40.9% (*n* = 9/22) of individuals with KS were discovered through means other than infertility checks, suggesting that a sampling bias might have affected the results. Reportedly, 4/33 (12%) of KS fetuses have testosterone levels at the level of girls during amniocentesis (13), and sensory and cognitive peculiarities may appear in KS in cases of early hypogonadism.

### 4.5 Is the extreme male brain theory appropriate?

In the present study, the higher tendency of sexual minorities assigned male at birth to exhibit phenotypes observed in ASD, and the negative associations of various indices with masculinity of the play in childhood suggested that, in males at birth, reduced early developmental androgen action may be associated with the phenotypes observed in ASD.

Although the EMB theory postulates that excessive androgen action during early development is a contributing factor to ASD (2, 3), negative data have also been reported, such as no association between testosterone in the amniotic fluid and ASD traits in either boys or girls (47), and no association between penile length or anogenital distance and ASD traits within male infants at birth and 3 months of age (48). The co-occurrence of ASD with various syndromes presenting with hypogonadism [for example, Prader-Willi syndrome (49) and Fragile X syndrome (50)] and an increase in ASD in conditions associated with low androgen levels have also been reported (51). Furthermore, there are reports of an association between ASD and KS or sexual minorities, including the present study, which assumes reduced androgen action during early development in males at birth. This is contrary to what would be expected from the EMB theory.

Considering the inconsistent results of findings both supporting and not supporting the EMB theory in individuals assigned male at birth, there may be a U-shaped instead of a linear association between early developmental sex steroids and ASD in males at birth. Moreover, early developmental sex steroid excess in periphery in males at birth may reflect an attempt to compensate for the reduced action of sex steroids in individuals through feedback. Furthermore, steroids are also produced in the brain, and neurosteroids, such as pregnanolone, bind allosterically to gamma-aminobutyric acid type A (GABA_A_) and *N*-methyl-D-aspartic acid (NMDA) receptors and may exert their physiological effects through non-genomic action without transcriptional regulation (52). For example, increased ASD-like behavior has been reported in male mice when pharmacology inhibits allopregnanolone synthesis (53). Therefore, the increase in sex steroids observed in the amniotic fluid and periphery may not necessarily reflect excessive action in the central nervous system, and steroidogenesis deficiency may be occurring in males at birth during early development.

### 4.6 Sex steroid deficiency and atypicality of sensory perception

Decreased early developmental sex steroid effects in males at birth may disrupt oxytocin function during the perinatal period and decrease the inhibitory effects in GABAergic neurons (8). Oxytocin neurons, like the hypothalamic paraventricular nucleus, are co-expressed with estrogen receptor beta (ERβ) and promoted by estrogen action (54). Oxytocin plays an important role for the prenatal transition of neuronal gamma-aminobutyric acid (GABA) neurotransmission from excitatory to inhibitory (GABA switch) (55). It is also suggested that prenatal exposure to endocrine disputing chemicals, such as bisphenol A or bisphenol S, hinders oxytocin function and GABA switch, likely by disrupting endogenous estradiol function (56). The disturbed GABA switch may contribute to ASD by increasing the excitation/inhibition (E/I) ratio and delaying synaptic pruning (57). It is assumed that failure of synaptic pruning causes localized hyperconnectivity in the brain, and an increase in the E/I ratio causes hypersensitivity in specific sensory areas. Furthermore, in mice with reduced electrophysiological signs of typical multisensory integration, GABAergic signaling in regions including the insular cortex was reduced, suggesting that it can be restored by pharmacologically enhancing GABAergic signaling during early development (58).

### 4.7 Gender dysphoria and atypicality of sensory perception and self-concept integration

Considering that gender dysphoric state was associated with phenotypes such as sensory hypersensitivity/hyposensitivity in the present study, individuals with GD may differ from neurotypical individuals in sensory processing, and thus in a part of self-concept formation, as in individuals with ASD. During sensory processing, the insular cortex and cingulate gyrus are involved in interoception, which is deeply involved in emotional processing (59). It is also the central region of the salience network that coordinates the switch between the default mode network (DMN) and the processing of the external world (60). Individuals with ASD show anatomical (61) and functional (62) changes in the insular cortex with low activity in the DMN while performing self-referential tasks and difficulty in forming self-concepts (63). Therefore, interoception may be involved in the atypicality of integrating self-concept and consciousness, as commonly observed in individuals with ASD.

Gender identity may also be regarded as a part of the self-concept that arises from a series of processes based on the external world and somatosensory self-references involving interoception, which involves the insular cortex. Individuals with KS (64, 65) and MTF transgenders (66) also have the reduced insular cortex. Although the estrogen receptors (ER) are not abundantly distributed in the insular cortex in rats, estrogen facilitates activation of descending sympathoexcitatory pathway by affecting GABAergic neurotransmission within the insular cortex (67). Functional connections from the right insular cortex to the left angular gyrus are increased in MTF transgenders treated with hormonal therapy (antiandrogen and estrogen administration) compared to cisgender men, and the alexithymia tendency is also altered by hormonal therapy (68). Furthermore, functional and structural differences have been suggested between transgender and cisgender individuals in the brain regions that process bodily sensations (69). Regarding bodily sensations, individuals with higher concentrations of salivary oxytocin may experience a stronger sense of body possession during the rubber hand illusion (70). Intranasal oxytocin also increases empathic appraisal and enhances functional connectivity between the right amygdala and insular and posterior cingulate cortices in response to positive stimuli (71). Although oxytocin receptors are also present in the insular cortex, their expression is more prominent in the anterior cingulate cortex and amygdala (72, 73), suggesting the importance of the insular cortex as a hub connecting oxytocin-regulated emotional networks.

### 4.8 Limitation

The present study had several limitations. First, unlike Baron-Cohen et al. (74), we did not use a strict definition of synesthesia (for example, that it is retained from childhood and is not caused by head injury or drugs), and the results were based solely on the participants’ self-reports, which require caution in interpretation. Second, the measurement of savant tendency made it possible to quantitatively measure the characteristics related to the abilities observed in savants, including the general population, rather than a dualistic interpretation of whether a person is a savant. However, since it is a self-report by participants, it must be conducted simultaneously with objective indicators, such as cognitive tests related to savant tendency, to ensure validity. Constructing an objective index of the savant ability itself is desirable. Third, although we requested cooperation from several hospitals and organizations to avoid sampling bias as much as possible, the sample sizes of KS and sexual minorities were small, and we could not eliminate the influence of sampling bias.

Despite these limitations, few studies have measured the sensory and cognitive characteristics of both individuals with KS and sexual minorities assigned male at birth and compared them with controls using matched random-sampling. This is a significant finding because it is an attempt to deal with indicators such as the savant tendency that may lead to the strengths of the participants. The effects observed in this study should be verified using various methodologies and sample groups.

## 5 Conclusion

The present study suggests that the prevalence of synesthetes and those with high tendencies to savant and sensory hypersensitivity/hyposensitivity are relatively common among sexual minorities assigned male at birth. Furthermore, gender dysphoric state and sensory hypersensitivity/hyposensitivity may be related to savant tendency and synesthesia. Our results suggest that GD may share a common physiological background with synesthesia, savant abilities, and atypical sensory sensitivity. Androgynous features (reduced sex steroid effects during early development) in males at birth may be partially related to phenotypes commonly observed in individuals with ASD. At least, the results do not rule out the possibility that both are partially related. In addition, the atypicality of a part of self-concept (identity), such as sexual minorities and gender dysphoria, may be particularly associated with the phenotype observed in individuals with ASD. We propose that the reduction of sex steroids during early development may lead to atypical neurodevelopment and disruption of the oxytocin and GABA systems, which may contribute to the atypicality of external and internal sensory perception, and thus to an atypicality of self-concept integration.

## Data Availability

The datasets presented in this article are not readily available because The data from this study was obtained from a special sample, and because there is a risk that individuals could be identified from the raw data, it cannot be made public due to ethical considerations. Requests to access the datasets should be directed to the corresponding authors.
