# Supplementary material for: Androgyny and atypical sensory sensitivity associated with savant ability: a comparison between Klinefelter syndrome and sexual minorities assigned male at birth
Source: Front Child Adolesc Psychiatry. 2024 Nov 8;3:1356802. doi: 10.3389/frcha.2024.1356802 (PMC11732014; doi:10.3389/frcha.2024.1356802)

Supplementary Material

Androgyny and atypical sensory sensitivity associated with savant ability: A comparison between Klinefelter syndrome and sexual minorities assigned male at birth

Shintaro Tawata*, Kikue Sakaguchi, Atsuko Saito

*** Correspondence:** Corresponding Author: [s-tawata-5g1@eagle.sophia.ac.jp](mailto:s-tawata-5g1@eagle.sophia.ac.jp)

# Exploratory analysis

## S1. Supplementary measures

### Savant tendency

The following questions were also asked; however, this study did not report the results. The respondents who answered “somewhat true” or “true” to the question “I can recognize the musical scale (do-re-mi...) just by listening to a sound” were asked whether they had been able to do so before receiving music education. Those who answered “somewhat true” or “true” to the question “I am a geek of some kind” were asked to answer freely what kind of geek they are. Finally, the respondents were asked to describe any characteristic episodes related to the content of each item or any skills or characteristics other than those listed above, if any.

### Masculinity of the play

Eight items preferred by boys: “monsters,” “computer games,” “miniature cars,” “toy guns,” “plastic models,” “baseball,” “soccer,” and “robots”

Eight items preferred by girls: “chatting,” “volleyball,” “drawing,” “rope jumping,” “stuffed animals,” “cooking,” “origami,” and “playing house”

## Supplementary results

## S2. Demographic data

| Variable | Level | KS  (*n* = 22) | Sexual minority  (*n* = 66) | Control 1^st^  (*n* = 36) | *p*-value | Multiple comparisons^d^ |
| --- | --- | --- | --- | --- | --- | --- |
| age^a^ |  | 41.50 ± 6.5 | 31.00 ± 9.00 | 44.00 ± 6.5 | < .001 | K>S, K=C, S<C |
| handedness^b^ | completely right-handed | 68.2 (15) | 71.2 (47) | 83.3 (30) | .083 |  |
|  | rather right-handed | 27.3 (6) | 16.7 (11) | 2.8 (1) |  |  |
|  | ambidexterity | 0 (0) | 3.0 (2) | 5.6 (2) |  |  |
|  | rather left-handed | 0 (0) | 6.1 (4) | 0 (0) |  |  |
|  | completely left-handed | 4.5 (1) | 3.0 (2) | 8.3 (3) |  |  |
| educational background^b^ | junior high school | 0 (0) | 1.5 (1) | 0 (0) | .002 |  |
|  | high school diploma certification | 4.5 (1) | 0 (0) | 0 (0) |  |  |
|  | high school | 9.1 (2) | 25.8 (17) | 5.6 (2) |  | S>C |
|  | junior college/ technical school/ technical college | 40.9 (9) | 9.1 (6) | 38.9 (14) |  | S<C |
|  | 4-year university | 36.4 (8) | 37.9 (25) | 41.7 (15) |  |  |
|  | 6-year university/ master's degree | 4.5 (1) | 16.7 (11) | 5.6 (2) |  |  |
|  | doctor's degree | 4.5 (1) | 9.1 (6) | 8.3 (3) |  |  |
| height^c^ |  | 176.55 ± 7.06 | 170.26 ± 5.39 | 170.22 ± 4.75 | < .001 | K>S, K>C, S=C |
| weight^a^ |  | 75.00 ± 7.50 | 60.50 ± 7.15 | 66.15 ± 8.90 | .003 | K>S, K=C, S=C, |
| body mass index^a^ |  | 24.00 ± 2.73 | 21.22 ± 2.59 | 22.57 ± 2.97 | .044 | K=S=C |
| mental illness^b^ | diagnosis | 31.8 (7) | 56.1 (37) | 2.8 (1) | < .001 | K=S, K>C, S>C |
| developmental disorder^b^ | diagnosis | 18.2 (4) | 30.3 (20) | 2.8 (1) | .004 | K=S, K=C, S>C |
| marital status with woman ^b^ | legal marriage | 68.2 (15) | 13.6 (9) | 61.1 (22) | < .001 | K>S, S<C |
|  | factual marriage/ cohabitation | 4.5 (1) | 4.5 (3) | 8.3 (3) |  |  |
|  | no | 27.3 (6) | 81.8 (54) | 30.6 (11) |  | K<S, S>C |
| *Note*. ^a^ Each cell represents median ± quartile deviation, with the *p*-value determined from the Kruskal-Wallis test. ^b^ Each cell represents % (*n*) in each group, with the *p*-value determined from Fisher's exact probability test. ^c^ Each cell represents mean ± SD, with the *p*-value determined from ANOVA. ^d^ K in each cell represents the KS group, S represents the sexual minority group, and C represents the control 1^st^ group. | | | | | | |

## S3. Diagnosis of mental illness

| Diagnosis | KS  (*n* = 22) | Sexual minority  (*n* = 66) | Control 1^st^  (*n* = 36) | *p*-value | Multiple comparisons |
| --- | --- | --- | --- | --- | --- |
| personality disorder | 0 (0) | 0 (0) | 0 (0) | ― |  |
| panic disorder | 9.1 (2) | 7.6 (5) | 0 (0) | .142 |  |
| anxiety disorder | 9.1 (2) | 10.6 (7) | 0 (0) | .078 |  |
| bipolar disorder | 4.5 (1) | 7.6 (5) | 0 (0) | .253 |  |
| depression | 9.1 (2) | 39.4 (26) | 2.8 (1) | <.001 | K<S, K=C, S>C |
| schizophrenia | 0 (0) | 1.5 (1) | 0 (0) | >.999 |  |
| obsessive-compulsive disorder | 4.5 (1) | 3.0 (2) | 0 (0) | .583 |  |
| addiction | 0 (0) | 1.5 (1) | 0 (0) | >.999 |  |
| *Note*. Each cell represents % (*n*) in the group, with the *p*-value determined from the Fisher's exact probability test. In multiple comparisons, K in each cell represents the KS group, S represents the sexual minority group, and C represents the control 1^st^ group. | | | | | |

In addition to the above, the following responses were received in the “Others” (free description) section.

KS: depressive state (*n* = 1)

sexual minority: adjustment disorder (*n* = 5), anthropophobia (*n* = 1), repetitive depressive disorder (*n* = 1), depressive state (*n* = 1), dysthymia (*n* = 1)

## S4. Diagnosis of other disorders

| Diagnosis | KS (*n* = 22) | Sexual minority (*n* = 66) | Control 1^st^ (*n* = 36) | *p*-value | Multiple comparisons |
| --- | --- | --- | --- | --- | --- |
| heart disease | 18.2 (4) | 4.5 (3) | 0 (0) | .016 | K=S=C |
| osteoporosis | 31.8 (7) | 0 (0) | 0 (0) | <.001 | K>S, K>C, S=C |
| gynecomastia | 31.8 (7) | 12.1 (8) | 0 (0) | <.001 | K>S, K>C, S>C |
| diabetes | 9.1 (2) | 1.5 (1) | 5.6 (2) | .153 |  |
| metabolic syndrome | 22.7 (5) | 3.0 (2) | 5.6 (2) | .013 | K>S, K=C, S=C |
| pneumothorax | 9.1 (2) | 3.0 (2) | 0 (0) | .159 |  |
| cryptorchidism | 4.5 (1) | 0 (0) | 0 (0) | .177 |  |
| micropenis | 9.1 (2) | 0 (0) | 0 (0) | .030 | K=S=C |
| erectile dysfunction | 18.2 (4) | 3.0 (2) | 2.8 (1) | .031 | K=S=C |
| sleep disorder | 36.4 (8) | 16.7 (11) | 2.8 (1) | .003 | K=S, K>C, S=C |
| epilepsy | 0 (0) | 1.5 (1) | 2.8 (1) | >.999 |  |
| autonomic ataxia | 18.2 (4) | 13.6 (9) | 0 (0) | .020 | K=S, K>C, S>C |
| tremor | 13.6 (3) | 1.5 (1) | 2.8 (1) | .053 |  |
| *Note*. Each cell represents % (*n*) in the group, with the *p*-value determined from the Fisher's exact probability test. In multiple comparisons, K in each cell represents the KS group, S represents the sexual minority group, and C represents the control 1^st^ group. | | | | | |

In addition to the above, the following responses were received in the “Others” (free description) section.

KS: dyspepsia (thin pancreatic duct) (*n* = 1), asthma (*n* = 1), suspected gynecomastia (*n* = 1)

sexual minority: hot flashes (*n* = 1), cerebral infarction scar (*n* = 1), fatty liver (*n* = 1), acute pyogenic osteomyelitis (*n* = 1), retinal detachment (*n* = 1), lung cancer (*n* = 1), inguinal hernia (*n* = 1), restless syndrome (*n* = 1), migraine (*n* = 1), suspected erectile dysfunction (*n* = 1), asthma (*n* = 2), allergies (*n* = 1), hepatitis b (*n* = 1)

## S5. Gender dysphoric state (yes(trans)/ other (nonbinary)/no)

| Gender dysphoric state | KS (*n* = 22) | Sexual minority (*n* = 66) | Control 1^st^ (*n* = 36) | *p*-value | Multiple comparisons ^c^ |
| --- | --- | --- | --- | --- | --- |
| other (nonbinary) | 13.6 (3) | 7.6 (5) | 0 (0) | < .001 |  |
| yes (trans) & yes | 18.2 (4) | 60.6 (40) | 0 (0) |  | K<S, K=C, S>C |
| *Note*. Each cell represents % (*n*) in the group, with the *p*-value determined from the Fisher's exact probability test. In multiple comparisons, K in each cell represents the KS group, S represents the sexual minority group, and C represents the control 1^st^ group. | | | | | |

## S6. KS subjects’ results by infertility/ other than infertility

### Gender dysphoric state

| Gender dysphoric state | Yes  (*n* = 4) | Other (nonbinary)  (*n* = 3) | No  (*n* = 15) |
| --- | --- | --- | --- |
| infertility (*n* = 13) | 0 | 2 | 11 |
| other than infertility (*n* = 9) | 4 | 1 | 4 |

### Savant tendency

| Savant tendency (Reanalysis) | Mean | SD |
| --- | --- | --- |
| infertility (*n* = 13) | 37.46 | 8.29 |
| other than infertility (*n* = 9) | 40.11 | 12.42 |

### GSQ total score

| GSQ | Mean | SD |
| --- | --- | --- |
| infertility (*n* = 13) | 40.15 | 17.97 |
| other than infertility (*n* = 9) | 61.00 | 34.03 |

### Diagnosis of developmental disorder

| Developmental disorder | No (*n* = 18) | Yes (*n* = 4) |
| --- | --- | --- |
| infertility (*n* = 13) | 12 | 1 |
| other than infertility (*n* = 9) | 6 | 3 |

*For those with a diagnosis of developmental disorder (*n* = 4), those who were infertile (*n* = 1) had a diagnosis of ADHD, and this person’s gender dysphoric state was other (nonbinary). Among the non-infertile individuals (*n* = 3), *n* = 1 had diagnoses of dysgraphia and dyscalculia, and this person’s gender dysphoric state was gender-neutral (before androgen administration). *n* = 1 had diagnoses of ASD, ADHD, and executive function disorder, and this person’s gender dysphoric state was other (nonbinary). *n* = 1 was other (nonbinary) with a diagnosis of ADHD.

*Despite not being included in the table, for infertile individuals, *n* = 1 who did not have a gender dysphoric state had “suspected dysgraphia,” *n* = 1 had “words do not come out immediately,” and this person’s gender dysphoric state was other (nonbinary).

### Diagnosis of ASD

| ASD | No (*n* = 21) | Yes (*n* = 1) |
| --- | --- | --- |
| infertility (*n* = 13) | 13 | 0 |
| other than infertility (*n* = 9) | 8 | 1 |

## S7. Synesthesia

Details of each participant's synesthesia are presented below. One participant in the sexual minority group (the person is nonbinary) responded that he/she “remembers/recalls colors associated with letters,” but this was not considered to be synesthesia.

| Group |  | Gender dysphoric state |  | Hormone administration |  | Synesthesia type |  | Changes with age and hormones^α^ | |
| --- | --- | --- | --- | --- | --- | --- | --- | --- | --- |
| KS^β^ |  | no |  | no |  | letter & taste →color/ season → smell |  | ― | |
|  |  | no^γ^ |  | yes |  | sound → picture |  | changes with hormones | |
| sexual minority |  | yes |  | yes |  | smell → color & sound/ expression & voice → color & light |  | no | |
|  |  |  |  |  |  | picture → sound |  | no | |
|  |  |  |  |  |  | music → vision |  | no | |
|  |  |  |  |  |  | sound → color |  | ― | |
|  |  |  |  |  |  | sound → color/ others |  | no | |
|  |  |  |  |  |  | number → color |  | no | |
|  |  |  |  |  |  | number → color |  | no | |
|  |  |  |  | no |  | letter → motion |  | changes with ages | |
|  |  |  |  |  |  | words → color |  | ― | |
|  |  |  |  |  |  | letter → color |  | changes with ages | |
|  |  |  |  |  |  | sound → color |  | ― | |
|  |  | other (nonbinary) |  |  |  | number & tone of music notation → color |  | ― | |
| control 1^st^ |  | no |  | no |  | emotion → vision |  | changes with ages | |
| *Note*. A row represents one person. ^α^ “―” in each cell indicates no specific description. ^β^ Two of the KS group members were found to have KS for reasons other than infertility. ^γ^ This participant was gender-neutral before androgen administration. | | | | | | | | |  |
|  |  |  |  |  |  |  |  |  | |

## S8. Savant tendency: two-way ANOVA (group (KS/sexual minority/control 1^st^) × gender dysphoric state (yes/no))

Since it is possible that the savant tendency differs depending on gender dysphoric state in addition to group differences, we conducted a two-way ANOVA by gender dysphoric state (yes/no) × group (KS/sexual minority/control 1^st^) and found a main effect of gender dysphoric state (*F* (1,119) = 2.21, *p* = .140, *η_p_^2^* = .02, 1 - β = .31 ), main effect of group (*F* (2,119) = 1.52, *p* = .223, *η_p_^2^* = .03, 1 - β = .32), and interaction (*F* (1,119) = 0.40, *p* = .842, *η_p_^2^* < .001, 1 - β = .05), none of which were significant.


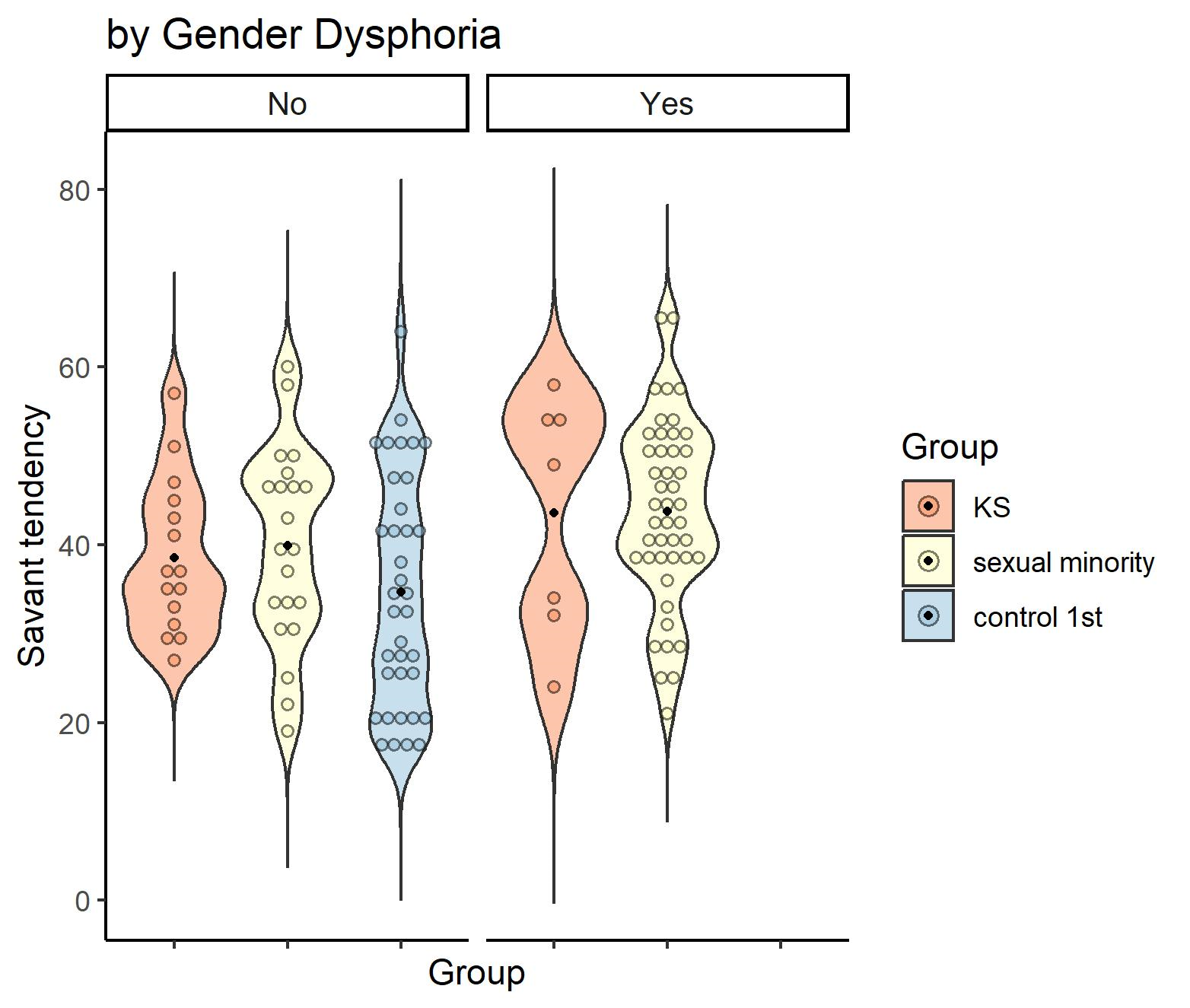


## S9. GSQ_ Means and standard deviations by modality and results of group comparisons

| Modality | KS (*n* = 22) | | | Sexual minority  (*n* = 66) | | | Control 1^st^ (*n* = 36) | | | *p*-value | Multiple comparisons^a^ |
| --- | --- | --- | --- | --- | --- | --- | --- | --- | --- | --- | --- |
| visual | 6.27 | ± | 4.21 | 7.14 | ± | 4.51 | 3.36 | ± | 4.06 | < .001 | K=S, K>C, S>C |
| auditory | 12.82 | ± | 5.84 | 13.65 | ± | 4.63 | 6.14 | ± | 5.38 | < .001 | K=S, K>C, S>C |
| olfactory | 7.32 | ± | 3.73 | 7.61 | ± | 3.70 | 3.53 | ± | 4.31 | < .001 | K=S, K>C, S>C |
| gustatory | 7.91 | ± | 4.28 | 6.98 | ± | 3.42 | 3.08 | ± | 3.64 | < .001 | K=S, K>C, S>C |
| tactile | 4.82 | ± | 4.71 | 5.95 | ± | 3.77 | 3.08 | ± | 3.82 | .003 | K=S, K=C, S>C |
| vestibular | 5.00 | ± | 4.98 | 6.32 | ± | 4.21 | 2.81 | ± | 3.34 | < .001 | K=S, K=C, S>C |
| proprioception | 4.55 | ± | 5.22 | 5.73 | ± | 4.12 | 3.14 | ± | 3.59 | .013 | K=S, K=C, S>C |
| *Note.* ^a^ K in each cell represents the KS group, S represents the sexual minority group, and C represents the control 1^st^ group. | | | | | | | | | | | |

## S10. GSQ: two-way ANOVA (group (KS/sexual minority/control 1^st^) × gender dysphoric state (yes/no))

Since it is possible that the total GSQ score differs depending on gender dysphoric state, in addition to group differences, we conducted a two-way ANOVA by gender dysphoric state (yes/no) × group (KS/sexual minority/control 1^st^) and found a main effect of gender dysphoric state (*F* (1,119) = 7.28, *p* = .008, *η_p_*^2^ = .06, 1 - β = .76) and a main effect of group (*F* (2,119) = 4.83, *p* = .010, *η_p_*^2^ = .08, 1 - β = .79). The interaction (*F* (1,119) = 0.098, *p* = .755, *η_p_*^2^ < .01, 1 - β = .06) was not significant (Figure 6).


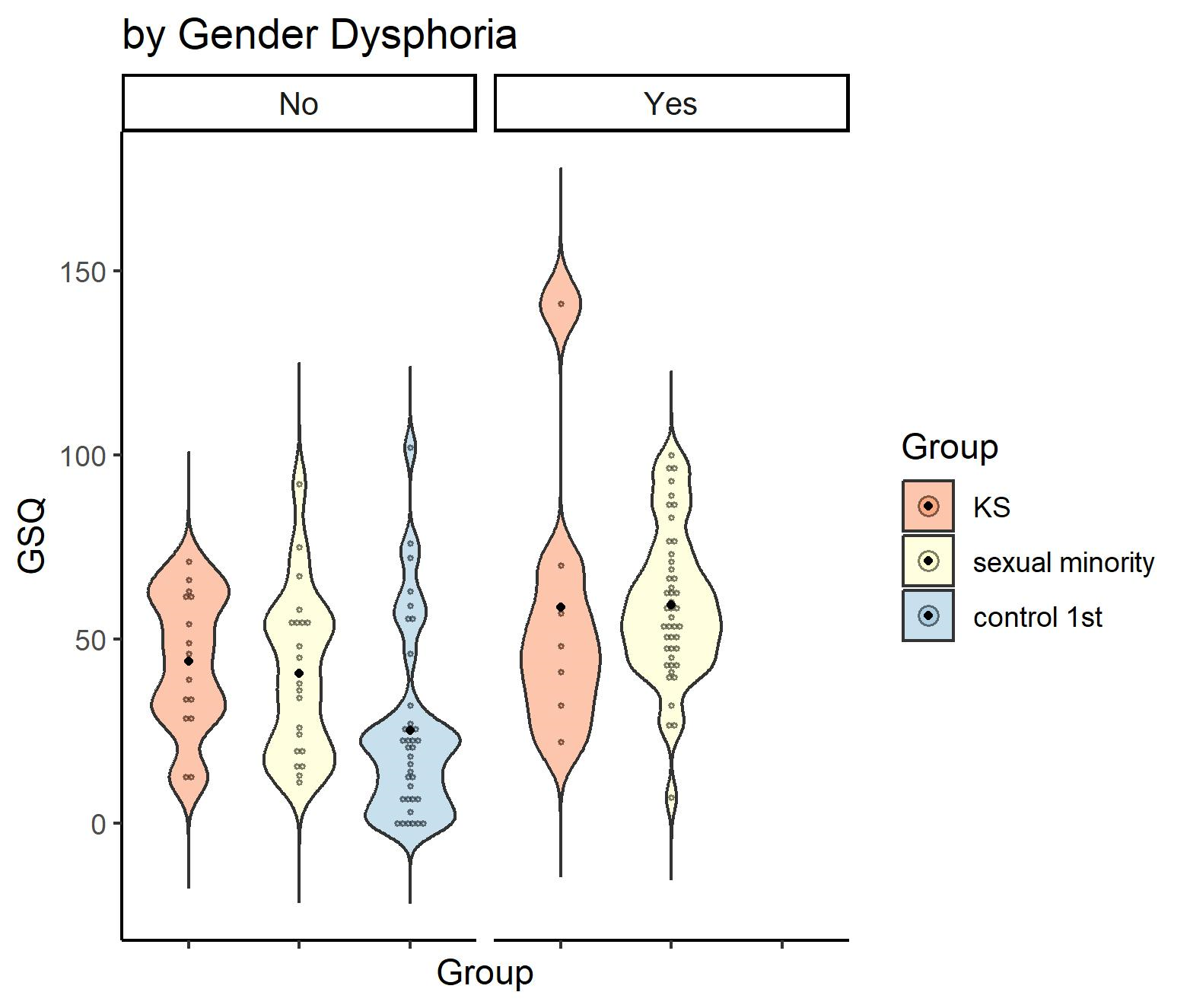


## S11. The model predicting savant tendency

| Dependent variable: savant tendency | β | *p*-value |
| --- | --- | --- |
| empathic sensitivity | .27 | .003 |
| handedness | .19 | .026 |
| gender dysphoric state | .19 | .032 |
| *R*^2^ (adjusted *R*^2^) | .19 (.17) |  |
| *F* (3, 120) | 9.15^***^ |  |
| ^***^*p* < .001 |  |  |

## S12. The model predicting GSQ

| Dependent variable: GSQ total score | β | *p*-value |
| --- | --- | --- |
| gender dysphoric state | .29 | .001 |
| diagnosis of developmental disorder | .23 | .008 |
| empathic sensitivity | .20 | .015 |
| *R*^2^ (adjusted *R*^2^) | .31 (.29) |  |
| *F* (3, 120) | 17.88^***^ |  |
| ^***^*p* < .001 |  |  |

# Reanalysis

## S13. Synesthesia (control 2^nd^)

For the control 2^nd^ group, the following are the descriptions that were judged as having synesthesia: “I felt gender in numbers when I was young,” “I feel certain colors and pain in my right arm against places where I should not go ahead,” “color in sounds,” “color in letters” (*n* = 2), “color in sounds until about age 10,” “feeling of being bent to certain sounds in techno music,” “color in smells and sounds from pictures,” “color in numbers/people,” “sometimes sounds seem to be 3D and letters appear to float.”

## S14. Association of savant tendency and GSQ by developmental disorder

Red, blue, and black indicate KS, sexual minority, and control 2^nd^, respectively. 〇 indicates no (not having a diagnosis of developmental disorder), and △ indicates yes (having a diagnosis of developmental disorder).


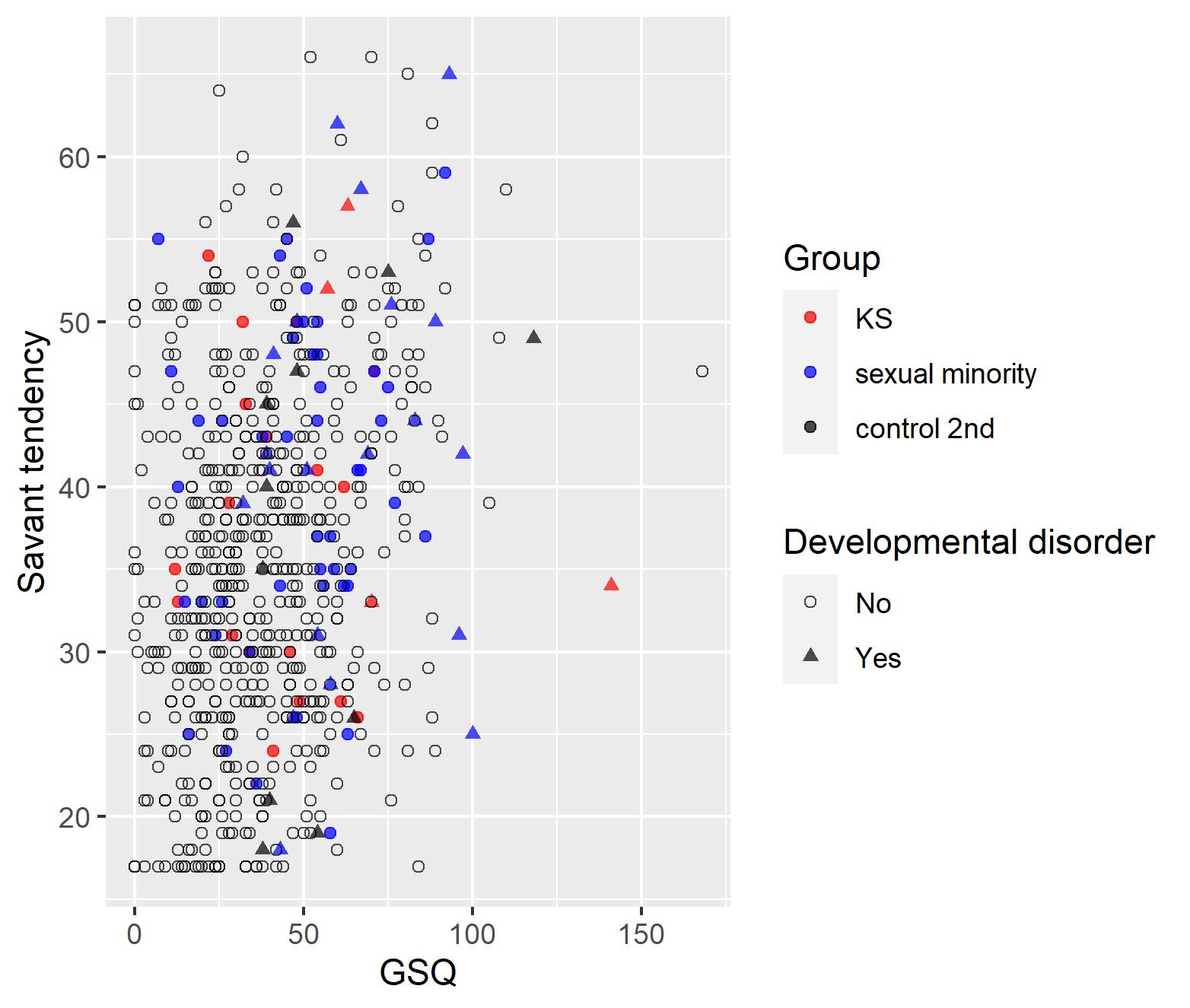


## S15. Correlation (*n* = 671)

| Spearman's ρ | a | b | c | d | e | f |
| --- | --- | --- | --- | --- | --- | --- |
| synesthesia: a |  |  |  |  |  |  |
| savant tendency: b | .072 |  |  |  |  |  |
| empathic sensitivity: c | .126** | .374** |  |  |  |  |
| gender dysphoric state: d | .304** | .153** | .265** |  |  |  |
| mental illness: e | .196** | .061 | .216** | .484** |  |  |
| developmental disorder: f | .171** | .091* | .193** | .483** | .441** |  |
| GSQ total: g | .181** | .233** | .305** | .241** | .221** | .214** |
| *Note:* ** significant at the 1% level, significant at the *5% level. “Synesthesia: a” is a dummy variable with values of “no=0, yes=1.” The “gender dysphoric state: d” is a dummy variable with “no=0, yes=1.” “Mental illness: e” is a dummy variable with values of “no=0, yes=1.” “Developmental disorder: f” is a dummy variable with values of “no=0, yes=1.” | | | | | | |

## S16. The model predicting savant tendency

| Dependent variable: savant tendency | β | *p*-value |
| --- | --- | --- |
| gender dysphoric state | .10 | .010 |
| GSQ total | .24 | < .001 |
| *R*^2^ (adjusted *R*^2^) | .08 (.07) |  |
| *F* (2, 668) | 27.82^***^ |  |
| ^***^*p* < .001 |  |  |

## S17. Additional analysis: regression including the presence or absence of a diagnosis of developmental disorder

### Dependent variable: synesthesia (no: 0, yes: 1)

predictor variable: gender dysphoric state (no: 0, yes: 1), diagnosis of developmental disorder (no: 0, yes: 1), GSQ total score

forced-choice method

χ²(2) = 40.863, *p* < .001

Nagelkerke *R²* = .223

・gender dysphoric state

Wald=20.064, *p* < .001, Exp(β)=10.366, 95%CI (3.726–28.839)

・developmental disorder

Wald=0.050, *p* = .823, Exp(β)=0.864, 95%CI (0.240–3.114)

・GSQ total

Wald=8.904, *p* = .003, Exp(β)=1.026, 95%CI (1.009–1.043)

### Dependent variable: savant tendency

predictor variable: gender dysphoric state (no: 0, yes: 1), diagnosis of developmental disorder (no: 0, yes: 1), GSQ total score

forced-choice method

adjusted *R^2^*= .073, Durbin-Watson = 1.900

*F* (3, 667) =18.524, *p* < .001

・gender dysphoric state

β = .102, *p* = .018

・developmental disorder

β = -.004, *p* = .922

・GSQ total

β = .236, *p* < .001

## S18. Additional analysis: logistic regression predicting gender dysphoric state

### Dependent variable: gender dysphoric state (no: 0, yes: 1)

predictor variable: synesthesia (no: 0, yes: 1), savant tendency, GSQ total score

variable increase stepwise method (based on likelihood ratio)

χ²(3) = 62.245, *p* < .001

Nagelkerke *R²* = .211

・savant tendency

Wald=5.312, *p* = .021, Exp(β)=1.035, 95%CI (1.005–1.067)

・synesthesia

Wald=23.117, *p* < .001, Exp(β)= 9.438, 95%CI (3.780–23.565)

・GSQ total

Wald=16.826, *p* < .001, Exp(β)=1.026, 95%CI (1.014–1.039)

## S19. Plots including LGBTs in Reanalysis

Score plots for four groups, including LGBT-identified individuals (*n* = 17), who were excluded from the control 2^nd^ group in Reanalysis.

### Savant tendency

Black dots indicate the mean values of each group.


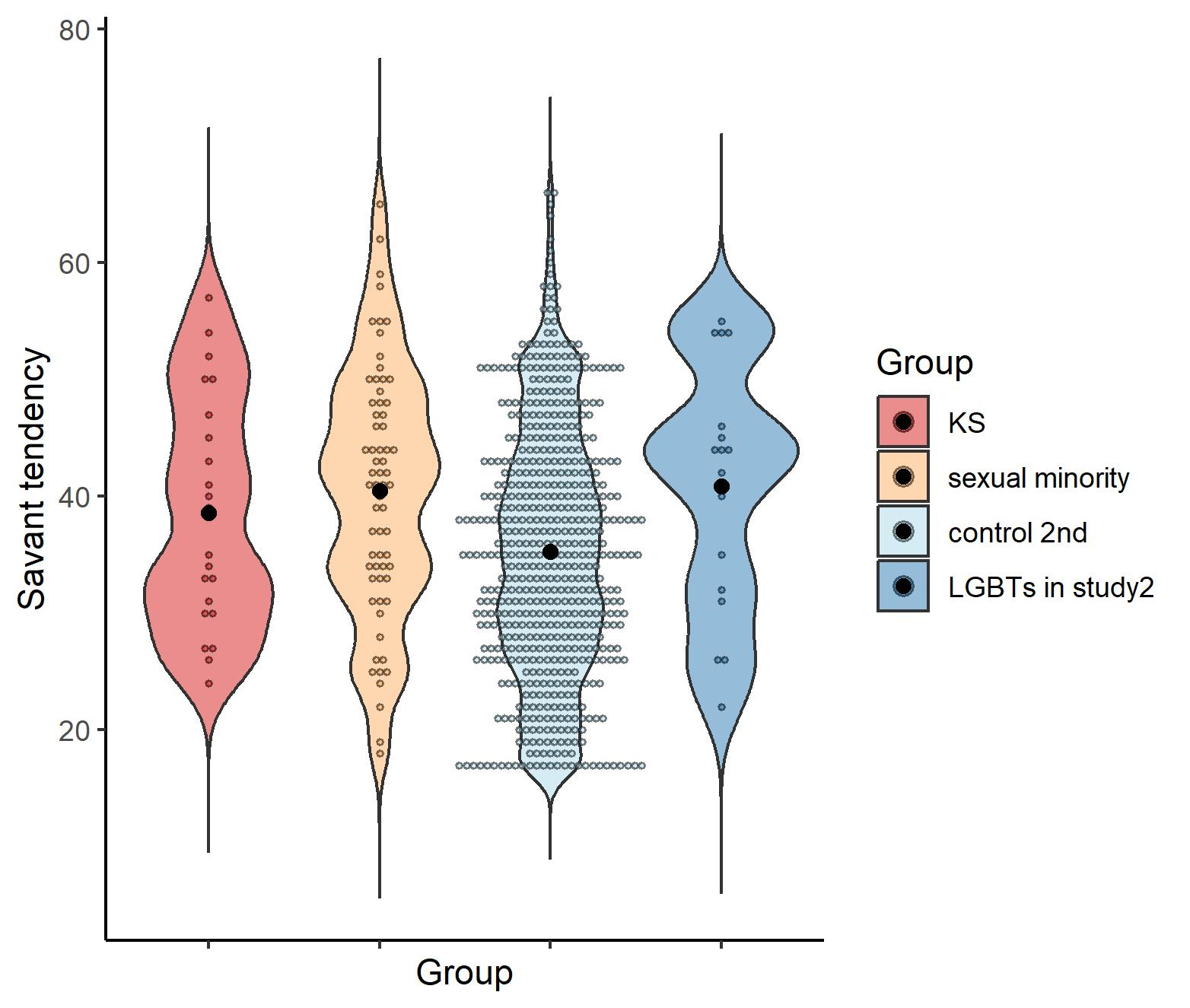


### GSQ

Black dots indicate the mean values of each group.


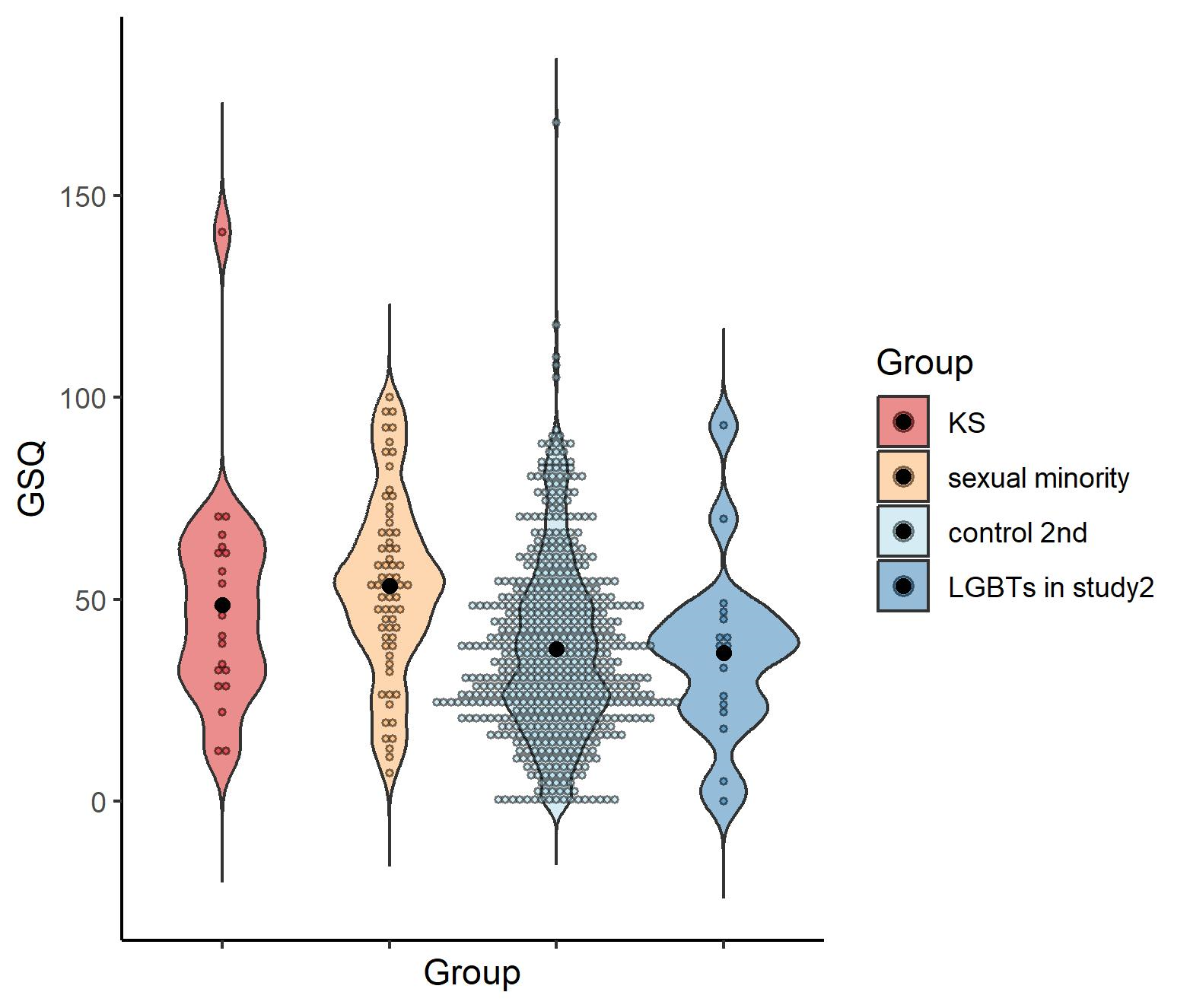

Supplement: Supplementary file 1 [file Datasheet1.docx]
